# Low mood, worry and mind wandering in children

**DOI:** 10.1111/bjdp.12561

**Published:** 2025-03-31

**Authors:** Ellen Teague, Teresa McCormack, Agnieszka J. Graham

**Affiliations:** ^1^ School of Psychology Queen's University Belfast Belfast UK

**Keywords:** child development, mind wandering, mood, temporal cognition, worry

## Abstract

Previous research with adults and adolescents has established that mind wandering—characterized by a shift in attention from external tasks to internal thoughts—is associated with negative affect and reduced psychological well‐being, particularly when focused on past events. This study explored the relations between low mood, worry, and the frequency and temporal orientation of mind wandering in children aged 8–12 years (*N* = 77). In a testing session conducted via videoconferencing software, we assessed mind wandering using intermittent thought probes during a listening activity and collected mood and worry data through self‐report questionnaires and carer reports. Our findings indicate that children's minds wandered approximately 23% of the time, aligning with existing literature. We found a significant association between lower mood and increased mind wandering. Specifically, children with lower mood showed a higher propensity for mind wandering. Moreover, low mood was a significant predictor of past‐oriented mind wandering, and a significant relation was observed between worry and future‐oriented thought. These results highlight the need for future research using experimental designs to clarify the causal relationships between children's mood, worry, and mind wandering. A deeper understanding of these dynamics is essential for developing targeted interventions that aim to enhance emotional well‐being in children.

**Statement of Contribution**

- Previous research has shown that mind wandering is associated with negative mood in adults and adolescents, and can also be measured in children.
- This study confirms that, similar to older populations, children aged 8‐12 with lower mood exhibit more frequent mind wandering.
- The findings further indicate that lower mood in children is a significant predictor of past‐oriented mind wandering.
- The study also provides preliminary evidence suggesting a relationship between worry and future‐oriented thoughts in children, warranting further exploration.

## INTRODUCTION

Mind wandering is a mental state in which attention to one's external surroundings is reduced in favour of internally generated thoughts, resulting in a state of perceptual decoupling from the here and now (Smallwood & Schooler, 2006). As an example, imagine arriving at your destination only to realize that you have not been paying attention for a long stretch of the drive, or missing key plot points in a movie because your mind was elsewhere. Indeed, adult studies of daily‐life mind wandering indicate that it consumes a substantial amount of time, estimated at 30%–50% (Killingsworth & Gilbert, 2010, but see Seli et al., 2018). While pervasive, instances of mind wandering are not necessarily problematic; rather, they can play an important role in the processing of personal goals and anticipating future events (e.g., Stawarczyk et al., 2011). However, it is also argued that mind wandering comes with an emotional cost—this assumption stems from extensive research showing that individuals prone to frequent mind wandering often experience heightened negative affect and diminished psychological well‐being (e.g., Deng et al., 2012; Killingsworth & Gilbert, 2010; Smallwood et al., 2007, 2009).

Although historically understudied, mind wandering in children is increasingly emerging as a crucial area of study. This shift is driven by recent demonstrations that it is possible to obtain meaningful momentary measurements of mind wandering in young children, and that mind wandering is linked to poorer learning performance (e.g., Cherry et al., 2022, 2023). To date, however, we know very little about how mind wandering relates to mood or well‐being in children. Given the limited research on this topic and considering the well‐documented links between mind wandering and mental well‐being in adults and adolescents (e.g., Marchetti et al., 2016; Vannucci et al., 2022), our study aimed to identify patterns of mind wandering associated with lower mood and worry in children aged 8 to 12 years. Investigating this link may provide valuable insights into whether mind wandering becomes dysfunctional at this developmental stage, help us detect early signs of emotional distress, and potentially contribute to the design of interventions that address negative mood states before they escalate.

### Mind wandering and mood in healthy and clinical adult and adolescent populations

The association between mind wandering and negative mood has been well established in typical adult and adolescent populations. These studies frequently use experience sampling whereby participants are periodically probed about whether their thoughts are on‐ or off‐task, either during daily life or in a laboratory task. In Killingsworth and Gilbert's (2010) experience sampling study, participants consistently reported lower happiness ratings during mind wandering episodes compared to periods of focused task engagement, even when their thoughts veered towards pleasant topics. These findings suggest that individuals are less happy when their minds wander, and time‐lagged analysis further indicated that mind wandering often preceded, rather than followed, feelings of unhappiness. However, further evidence suggests that the relation between mind wandering and mood is more complex, as mind wandering can both trigger and result from negative mood. Ruby et al. (2013) reported that mind wandering was associated with mood deterioration, but this effect was observed only when a positive mood preceded mind wandering. They also found that individuals experiencing low mood reported higher levels of mind wandering. To further explore the causal link between mind wandering and mood, Poerio et al. (2013) examined the temporal sequence of sadness and anxiety in relation to mind wandering episodes in daily life. They found that while feelings of sadness often preceded mind wandering, the act of mind wandering itself did not necessarily lead to subsequent sadness or anxiety (see also Franklin et al., 2013).

A complementary strand of research involving clinical populations has revealed a higher prevalence of mind wandering among individuals with elevated depressive symptoms (e.g., Deng et al., 2012; Smallwood et al., 2007), including in adolescent populations (Fredrick & Becker, 2020; Vannucci et al., 2020). Although correlational, these findings suggest that mind wandering may exacerbate mood disturbances, initiating a cognitive cycle where increased mind wandering further deteriorates mood, potentially intensifying depressive symptoms (Nayda & Takarangi, 2021). This cycle often involves rumination, characterized by a rigid and repetitive focus on distress (Nolen‐Hoeksema, 1991). It is also plausible that mind wandering could be a consequence of low mood, rather than a causal antecedent, or that the relationship could be bidirectional, as we discuss later. By contrast to the more extensive literature on mind wandering and depression, few studies have examined its relation to other forms of emotional distress, though some noteworthy findings have emerged. For instance, a positive correlation was found between reported levels of anxiety and instances of mind wandering (Figueiredo et al., 2020; Seli et al., 2019). Mind wandering has also been associated with the construct of worry—defined as an attempt at mental problem‐solving regarding future concerns—particularly when characterized by perseverative (Makovac et al., 2019), unintentional (Seli et al., 2019), excessive (Figueiredo & Mattos, 2021), or negatively valenced but future‐oriented thoughts (Vannikov‐Lugassi & Soffer‐Dudek, 2018).

### Mood and temporal orientation of mind wandering

Studies that clearly distinguish between mind wandering to the future versus the past―i.e., that have measured the temporal orientation rather than just the frequency of mind wandering―have helped shed further light on the nature of the associations between mood and mind wandering. Adults typically tend to mind wander more towards the future than the past, i.e., show ‘prospective bias’ (e.g., Smallwood & Schooler, 2015; Stawarczyk, 2018). Future‐oriented thoughts have been associated with enhanced problem‐solving, decision‐making, and emotional regulation, and are generally linked to more positive emotions (e.g., Baird et al., 2011; D'Argembeau et al., 2011). However, while future‐oriented mind wandering is generally regarded as adaptive and beneficial for planning and goal setting (Suddendorf & Corballis, 2007), excessive worry—despite being future‐oriented—can become uncontrollable and disruptive, worsening mood and heightening anxiety (e.g., Calmes & Roberts, 2007; Makovac et al., 2019). Taken together, these findings underscore the nuanced balance between goal‐directed mind wandering, which can be beneficial, and excessive worry, which can be detrimental to mental health.

Mind wandering about the past has been found to have distinct patterns and associations with mood and mental health that differ from those found for mind wandering to the future. Generally, thoughts about the past have been linked to more negative mood states. For instance, Smallwood and O'Connor (2011) found that the induction of a sad mood increased retrospective mind wandering, suggesting that negative emotions may bias mind wandering towards past events. Experience sampling studies further support this connection in both adults and adolescents (Poerio et al., 2013; Webb et al., 2021, 2022). Importantly, while negative mood can trigger past‐oriented thoughts, these thoughts can also perpetuate negative mood (Ruby et al., 2013). Additionally, research indicates that depressed individuals are more prone to engaging in past‐oriented thoughts during mind wandering (Hoffmann et al., 2016; McKay et al., 2017). In summary, the link between mood and mind wandering appears to vary by temporal orientation. Mind wandering to the past is generally associated with low mood, whereas mind wandering to the future can be positively associated with mood, with the caveat that excessive worry about the future, which is sometimes described as a sub‐type of future‐oriented mind wandering (Fell et al., 2023), may be detrimental to well‐being. Both in the case of past‐ and future‐oriented mind wandering, two‐way causal relations can hold between mood and mind wandering (i.e., mood can influence mind wandering, and mind wandering can influence mood).

### Mind wandering in children

Recent research into mind wandering in children has grown rapidly, revealing its prevalence during childhood with estimates ranging from 20% to 33% (e.g., Cherry et al., 2022; Keulers & Jonkman, 2019; Ye et al., 2014; Zhang et al., 2015). Various methods have been employed to study mind wandering in this age group. Some studies adopt self‐report questionnaires (e.g., Cao et al., 2022; Frick et al., 2019), while others apply experience sampling methods—specifically ‘probe‐caught’ techniques—that have proven effective with children. Probe‐caught studies typically use visual and auditory prompts intermittently while participants are completing a separate laboratory task, with participants asked to reflect on the contents of their thoughts and report whether, at the time of the probe, they were focused on the task or mind wandering. Children have demonstrated their ability to reliably distinguish between task‐related and task‐unrelated thoughts across a range of such tasks (e.g., Cherry et al., 2022, 2023; Keulers & Jonkman, 2019; Zhang et al., 2015).

Several studies have explored the temporal orientation of mind wandering in children, but the results have been inconsistent. Zhang et al. (2015) found that children aged 9–11 years reported more future‐oriented than past‐oriented mind wandering during a Go/No‐Go task; Ye et al.'s (2014) results are similar in suggesting the presence of a prospective bias in mind wandering by late childhood. However, this conclusion is challenged by more recent results. McCormack et al. (2018) did not find a clear prospective bias among children aged 6–7 and 9–10, or among adolescents aged 14–15 years. Although a consistent future‐oriented bias was not observed, McCormack et al.'s findings suggest that while a future‐oriented focus may not be pervasive in younger populations, there is evidence of an increase in future‐oriented thoughts with age, indicating that the temporal orientation of mind wandering may change as children develop.

Very little research has directly examined the relation between mood and mind wandering in children. Jiang et al.'s (2021) studied retrospective self‐assessments of the temporal orientation of mind wandering across two very large cohorts of children and adolescents aged 11–18 years. Their findings indicated a positive association between both past‐ and future‐oriented thoughts and overall well‐being, which on the face of it contradicts similar findings in adolescents and adults, particularly regarding past‐oriented thoughts. However, we note that their measure of levels of past‐ and future‐oriented thoughts involved rating quite specific statements (e.g., ‘Childhood playmates often suddenly appear in my mind.’) that do not seem to function as straightforward measures of the frequency of past or future mind wandering. Interpreting the findings of this study thus highlights the need to carefully consider the nature of any self‐report questionnaires used to measure mind wandering.

The aim of a study by Jin et al. (2022) was to examine if mind wandering mediated the relation between mindfulness and emotions in children. In this study, participants answered questions about whether they had been inattentive or ‘in a daze’, which the researchers interpreted as measuring mind wandering, and about their affective states each day over ten days. All participants attended elementary school, although the age range was relatively wide (9–14 years) meaning the sample included some adolescents. This study had mixed findings, insofar as no relations were found between individuals' day‐to‐day reports of mindfulness, mind wandering, and emotional states (i.e., varying levels of emotions each day did not track the day‐to‐day varying levels of mindfulness and self‐reported mind wandering). However, in a between‐subjects analysis, participants' average levels of mind wandering across the ten days were positively associated with average self‐reported negative emotions and negatively associated with positive emotions. Modelling of the data revealed that mindfulness predicted greater positive emotions and reduced negative emotions through lower levels of mind wandering. However, this mediation effect was not observed at the 6‐month follow‐up.

A more recent study by Hoffmann et al. (2023) investigated thought patterns in children aged between 6 and 12 years, with and without a history of childhood maltreatment, during a non‐demanding letter discrimination task with embedded thought probes. This study used a relatively complex method of measuring thought patterns whereby, each time they were probed, children made judgements about their thoughts on a 9‐point Likert scale across 7 different dimensions (off‐task, positive valence, negative valence, self‐related, other‐related, past‐oriented, future‐oriented). The authors found no group differences in the ratings regarding the extent to which thoughts were off‐task, nor in the ratings of past‐ or future‐orientation, although they did find that maltreated children rated their thoughts as being less positive. The main difference between the groups was in the detailed pattern of associations between children's ratings on the seven dimensions (see Hoffman et al. for details); nevertheless, higher ratings of past‐orientation were associated with depressive symptoms in the maltreated group.

### Current study

As things stand, it is difficult to draw any clear conclusions about associations between the frequency and temporal orientation of mind wandering and mood in children, and whether they are similar to those observed in adults or adolescents. Jin et al. (2022) and Hoffmann et al. (2023) suggest some connections, but we note that both of these studies used measures that are not straightforward to interpret as measures of mind wandering frequency per se (indeed, Hoffman et al. were interested in thought patterns indicative of rumination rather than mind wandering). As far as we can see, the studies also did not check to what extent children (who were as young as 6 years) actually understood the potentially difficult meta‐cognitive judgements they were being asked to make.

The current study addressed the existing gap in the developmental literature by examining the association between the frequency and temporal focus of mind wandering and a range of indices of mood and well‐being among typically developing children aged between 8 and 12. It used a well‐established probe‐caught technique, which is particularly beneficial with children in that it does not require continuous monitoring of the contents of the mind. Instead, it captures task‐unrelated thoughts periodically through probes (e.g., *What were you thinking about just now*?), providing real‐time data on mind wandering and mitigating the biases associated with retrospective self‐reports. In adult populations, this type of experience sampling has been shown to correlate with physiological markers of mind wandering (Reichle et al., 2009) and with questionnaire‐based reports (Mrazek et al., 2013). Moreover, the particular thought probe method used has been shown to be effective in assessing the impact of mind wandering on cognitive processes, such as memory, in children as young as 6 years old (Cherry et al., 2022, 2023).

We formulated three hypotheses for the study. First, drawing from the literature on adults and adolescents, we predicted that lower mood would be associated with higher rates of mind wandering. Second, we hypothesized that the temporal orientation of mind wandering would be linked to mood, specifically anticipating a positive correlation between past‐oriented thoughts and lower mood. Finally, in the light of the research on worry and mind wandering, we tentatively predicted that levels of self‐reported worry would be associated with the frequency of future‐oriented thoughts.

## METHOD

### Participants

The total sample included 80 children (46 female) aged between 8 and 12 years. Three participants were excluded from the analysis due to incomplete data sets (1 due to internet connectivity issues and 2 due to reluctance to complete the mind wandering task), reducing the sample size to 77 (*M*_age_ = 9.75, *SD*_age_ = 1.29). All children were living in the United Kingdom or the Republic of Ireland. Those reported by their parents to have been diagnosed with neurodevelopmental disorders or mental health conditions were excluded from the sample. Participants were recruited through parental interest generated by social media advertisements and snowball sampling, with a £50 voucher raffle to increase interest in the study.

Post hoc analysis of the achieved sample size for conducting a linear regression containing two predictors, assuming a medium‐sized effect (*f* ^2^ = 0.15) and α of .05, was calculated using G*Power (version 3.1; Faul et al., 2009) and yielded a power of 0.86.

### Materials

#### Self‐reported measures of mood

The Stirling Children's Well‐being Scale (SCWBS; Liddle & Carter, 2015), designed for children aged 8 to 15 years, offers a holistic assessment of emotional and psychological well‐being over the weeks prior to testing. The scale consists of 15 positively worded items, such as ‘I've been feeling calm,’ rated on a 5‐point Likert scale. Children are instructed to rate their answers based on their thoughts and feelings over the past several weeks. The SCWBS scores range from 12 to 60, with higher scores indicating better well‐being. The scale contains three subscales: positive emotional state (6 items), positive outlook (6 items), and social desirability (3 items). For the current study, the positive emotional state and positive outlook subscales were combined to create a measure of well‐being. Previous research has demonstrated that the SCWBS has good internal reliability, with a Cronbach's alpha of .8, which is consistent with the unidimensional measure of reliability obtained in the current study (α = .77). The SCWBS also showed good convergent validity, as evidenced by its correlations with established measures of well‐being and self‐esteem (Liddle & Carter, 2015; Nishida et al., 2021).

The Positive and Negative Affect Schedule for Children (PANAS‐C; short form; Ebesutani et al., 2012) assesses mood states in children aged 6–18. This measure comprises 10 items divided into positive (joyful, cheerful, happy, lively, proud) and negative affect (miserable, mad, afraid, scared, sad) scales. Children rate the frequency of these emotions experienced over the past few weeks on a 5‐point Likert scale, with higher scores reflecting higher levels of either positive or negative affect. Scores on both scales range from 5 to 25. The short form PANAS‐C has demonstrated strong internal reliability, with Cronbach's alpha values of .86 and .82 for positive and negative items, respectively (Ebesutani et al., 2012). In the current study, the Cronbach's α statistics were slightly lower, at .71 for the positive affect scale and .72 for the negative affect scale. The validity of the short form PANAS‐C has been demonstrated in terms of its ability to provide clinically useful information (Ebesutani et al.) and its correlations with established measures of anxiety and depression (Wróbel et al., 2019).

The Child Shortened Mood and Feelings Questionnaire (CSMFQ, short version; Angold et al., 1995; Costello & Angold, 1988) serves as a screening tool for depression in children and young people aged 6 to 19 years. It includes 13 descriptive phrases, such as ‘I cried a lot,’ and asks children to rate how they have been feeling or acting during the past few weeks. Responses are given on a 3‐point scale: ‘not true’, ‘sometimes true’ and ‘always true’. Scores range from 0 to 26, with a score ≥ 12 indicating potential depression. The CSMFQ has demonstrated good internal reliability (Cronbach's α ranging from .85 to .89; Jarbin et al., 2020; Kuo et al., 2005; Thabrew et al., 2018) and its validity has been established through its ability to discriminate between children who have and have not been diagnosed with depression (Angold et al., 1995). In the current study, the Cronbach's alpha value was .69, suggesting moderate reliability.

#### Carer‐reported measures of mood

Positive and Negative Affect Schedule for Children – Parent's version (PANAS‐C‐P; shortened version; Ebesutani et al., 2011; Watson et al., 1988) was used to measure carer‐reported mood. This version mirrors the child version, covering five positive and five negative emotional states, and uses the same 5‐point scale. The shortened PANAS‐C‐P has demonstrated good internal reliability, with Cronbach's alpha values of .85 for positive affect and .83 for negative affect reported by Ebesutani et al. (2011), and .86 and .76, respectively, in the present study.

The Parent Shortened Mood and Feelings Questionnaire (PSMFQ; parent‐rated short version; Angold et al., 1995; Costello & Angold, 1988) is a carer‐reported screening tool for depression in children and young people. It includes 13 descriptive phrases identical to those in the child‐rated MFQ, but from the carer's perspective, for example: ‘they cried a lot’. Carers rate their child's mood on a 3‐point scale. Scores range from 0 to 26, with higher scores indicating greater depressive symptoms. The PSMFQ has demonstrated good internal reliability, with Cronbach's alpha values between .85 and .89 in previous reports (Jarbin et al., 2020), and .80 in the current dataset.

#### Self‐reported measure of worry

The Penn State Worry Questionnaire for Children (PSWQ‐C; Chorpita et al., 1997) is a self‐report measure designed to assess the tendency of children and adolescents aged 8–17 years to engage in excessive and uncontrollable worry. It consists of 14 items, such as “my worries really bother me.” Respondents rate the extent to which each statement applies to them using a 4‐point scale, ranging from “never true” to “always true.” Total scores on the PSWQ‐C range from 0 to 42, with higher scores indicating a greater tendency to worry. The PSWQ‐C has good internal reliability, with a Cronbach's α of .82, as reported by Muris et al. (2001), and .87 found for the current sample. Its validity has been demonstrated through its correlations with existing measures of worry and low mood, as well as its ability to discriminate between children with clinically confirmed anxiety disorders and those without such disorders (Chorpita et al., 1997).

#### Mind wandering task

The mind wandering task was delivered via a Microsoft PowerPoint presentation, with both training activities and experimental tasks accompanied by audio narration to ensure accessibility. The first training activity aimed to familiarize children with the concepts of ‘past’, ‘future’, and ‘now’. Three different scenarios were presented, each describing activities taking place in one of these timeframes (e.g. “Timmy rode his bike yesterday”). Children were required to identify the correct time orientation of each activity to proceed to the next training phase. All participants achieved 100% accuracy across the three trials for this task.

The second training activity introduced children to the concepts of on‐task and off‐task thoughts, as well as thoughts about the past, present, and future. During this activity, children listened to a brief description of a cartoon character's thoughts before responding to thought probes. For each of the four thought probes, participants first determined whether the cartoon character's thoughts were on‐task (“thinking about what was just said in the story”) or off‐task (“thinking about something other than the story”). If the character was off‐task, participants had to categorize whether the thought was oriented to the past (“something that has happened”), oriented to the future (“something that will happen”), or anchored in the present (“here‐and‐now”). If participants made two incorrect responses across the four trials, they completed additional training consisting of four extra practice trials. Only two participants required this additional training. The precise wording of all task procedures can be found in the Supplement.

**FIGURE 1:**
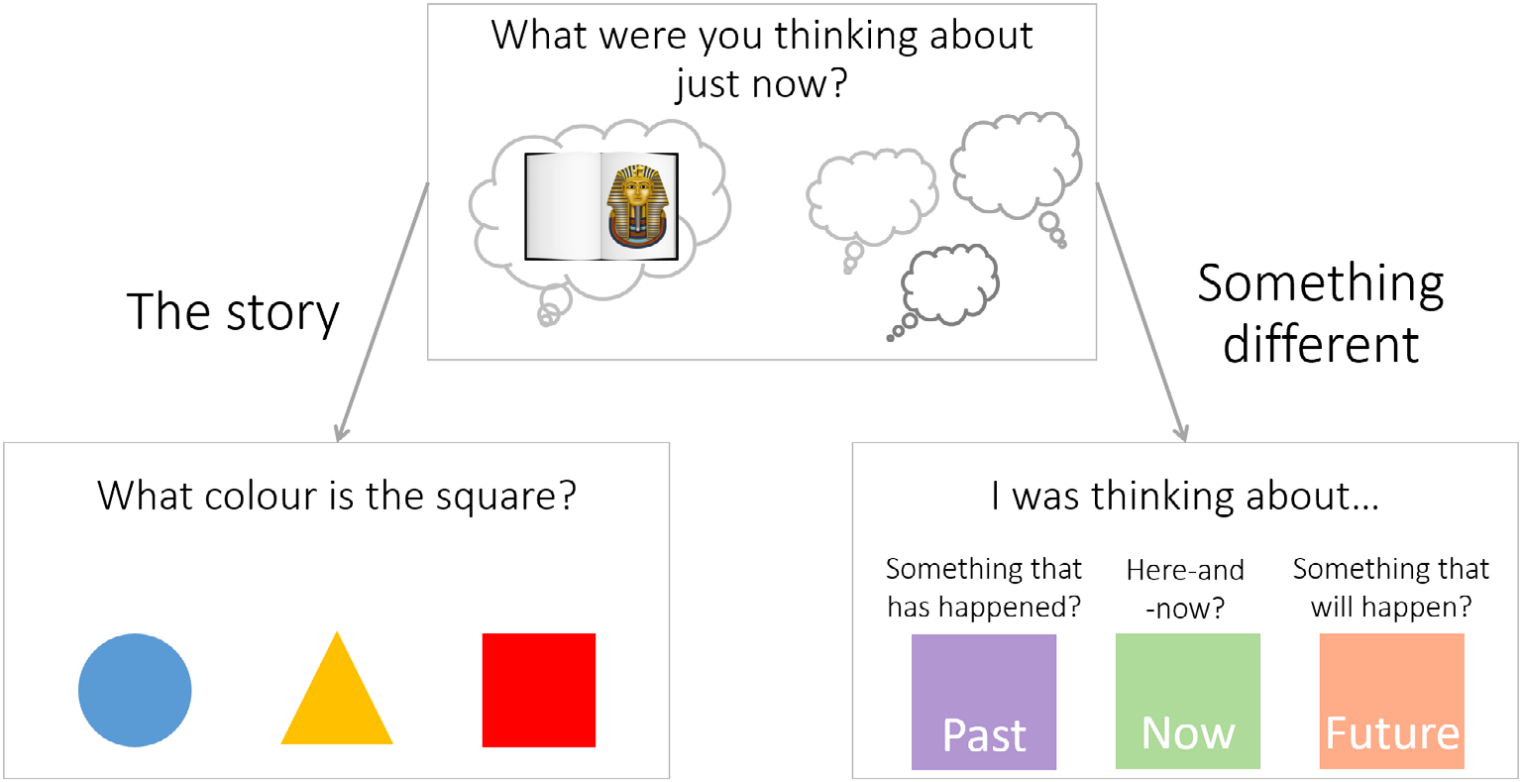
Thought probe example with possible responses.

On successful completion of the training activities, children progressed to the main task, where they listened to a story about ancient Egypt and responded verbally to probes about their thoughts. The story was 12 min long and adapted from Cherry et al. (2022, 2023). Following recommendations from Welhaf et al. (2023), eight intermittent thought probes were embedded in the story and presented visually (Figure 1). Each probe appeared on the screen with an accompanying ‘ding’ sound approximately every 90 s (range: 80–105 s). At the beginning of each probe, participants were presented with an initial question to determine whether their thoughts were on‐ or off‐task (e.g., “What were you thinking about just now?”). Participants could choose between two possible answers: “what was just said in the story” or “something else.” If participants indicated that their thoughts were off‐task, they were prompted to categorize them as either past‐oriented, future‐oriented, or related to the present moment. Conversely, if participants indicated that they were thinking about the story, they were shown an image of three different coloured shapes and asked to name the colour of one of the shapes (e.g. “What colour is the square?”). This factual question was included in an attempt to standardize task completion times.

### Procedure

Parents consented to their child's participation via an online questionnaire and guided their child through a simplified assent form. Child assent was reaffirmed at the start of the session, where key study details were presented in a child‐friendly manner, and participants were reminded of their right to withdraw at any time. Each testing session was conducted over Microsoft Teams videoconferencing software and lasted approximately 30 min. The session began with the researcher providing an age‐appropriate overview of the study. Subsequently, self‐report measures of mood and worry were administered. The researcher guided this process by verbally presenting instructions and questions for each measure to the child, while also displaying visual aids with the questions and available response options. These were presented in large font, with one question displayed at a time to enhance clarity (further details are available in the supplement and on the OSF). Children provided their responses verbally throughout the session, with any ambiguous responses clarified through prompts from the experimenter. Each child then participated in the two training activities, followed by listening to the story about Egypt and responding to eight intermittent thought probes. During the sessions, parents were absent from the room where the child was on the video call 53.2% of the time (*n* = 41). In 20.8% of cases (*n* = 16), the parent sat beside the child, and in 26.0% of cases (*n* = 20), the parent was in the background. This aspect of the procedure (parent presence or absence during testing) was not controlled for but was recorded by the experimenter. A Kruskal‐Wallis test showed that the reported mind wandering rate did not vary by parent presence (*p* = .172, BF_10_ = 0.56), and neither did the mood composite (*p* = .936, BF_10_ = 0.13).

In advance of the testing sessions, parents and caregivers received a link to an online survey containing carer‐related measures, which could be completed either before or within a few days after. However, the interval was not controlled for, and this is discussed as a possible procedural limitation later in the manuscript. Each parent or caregiver was provided with a unique ID for their child, enabling them to access and complete questionnaires online.

### Statistical analysis

Data were analysed using JASP (Version 0.9.1; JASP Team, 2024). Both the data files and the JASP analysis files are available on the Open Science Framework [https://osf.io/2a9ze/]. We supplemented traditional null hypothesis significance testing (NHST) with Bayesian statistics, which some argue provide a more robust basis for probabilistic inference (e.g., Kruschke, 2011). Bayesian methods also allow for the quantification of evidence for the null hypothesis, offering evidence for the absence of an effect, rather than merely the absence of evidence for an effect, as is the case with NHST. We use the notation BF_10_ to denote the Bayes factor (BF) for the presence of an effect. While a BF of 1 is typically considered indicative of no evidence, a BF between 1 and 3 is considered to provide anecdotal evidence, and a BF greater than 3 is considered to provide substantial evidence (Jeffreys, 1961; Wetzels & Wagenmakers, 2012). However, these labels are subjective and should be applied cautiously, with readers encouraged to assess the strength of evidence themselves.

To test the hypotheses outlined in the Introduction, we conducted regression analyses, preceded by zero‐order correlations to inform the selection of predictors for the regression models. These analyses were conducted using generalized linear models in JASP, employing a Binomial family distribution, which is appropriate for modelling proportional data such as mind wandering rates. The default logit link function was used to facilitate the interpretation of parameter estimates. Age was included as a covariate in our models, given its potential influence on mind wandering patterns. While the developmental trajectory of mind wandering in childhood is not well understood, research on older adults suggests that age may affect mind wandering patterns (e.g., Frank et al., 2015; Maillet et al., 2018), and Cherry et al. (2023) discuss findings that suggest levels of mind wandering may change developmentally over childhood. Therefore, age was included in the null model to account for its potential impact. Predictor selection and other inferences were based on *p* values, with Bayes factors (BFs) included for transparency and completeness.

## RESULTS

Table 1 provides the average self‐reported and carer‐reported mood scores, along with rates of mind wandering. Overall, children in the sample reported mind wandering 23.1% (*SD* = 18.7%) of the time: 8.0% (*SD* = 9.9%) about the future, 7.4% (*SD* = 10.1%) about the past, and 8.3% (*SD* = 10.4%) about the present. A chi‐square analysis revealed no significant difference between the frequency of future‐ and past‐related thoughts (*χ* ^2^(9) = 10.21, *p* = .333, BF_10_ = 0.05). Independent samples t‐tests (Table S1 in the Supplement) revealed a significant gender effect only for SCWBS social desirability scores, with girls (*M* = 11.41, *SD* = 2.07) scoring higher than boys (*M* = 9.58, *SD* = 2.01; Mann–Whitney *U* = 1047.00, *p* < .001, BF_10_ = 100.91).

**TABLE 1:** Self‐reported and carer‐reported measures of well‐being and mind wandering rates.

	*N*	Mean (*SD*)	Skewness	Kurtosis
Self‐reported measures of well‐being				
SCWBS	77	46.69 (5.00)	−0.72	0.51
SCWBS (social desirability score)	77	10.68 (2.23)	0.02	1.84
CSMFQ	77	4.73 (3.11)	0.97	1.54
PANAS‐C negative affect	77	8.81 (3.19)	0.73	−0.31
PANAS‐C positive affect	77	20.08 (3.15)	−0.55	−0.34
Self‐reported measure of worry				
PSWQ‐C	77	15.87 (7.44)	0.56	−0.11
Carer‐reported measures of well‐being				
PANAS‐C‐P negative affect	69	8.67 (2.89)	1.02	1.28
PANAS‐C‐P positive affect	69	20.54 (3.23)	−0.53	−0.09
PSMFQ	69	3.81 (3.98)	2.21	7.28
Mind wandering				
Total	77	1.86 (1.53)	0.47	−0.79
Past	77	0.57 (0.80)	1.08	−0.05
Present	77	0.65 (0.82)	1.03	0.14
Future	77	0.62 (0.78)	1.12	0.72

*Note*: Means and SDs for mind wandering (total, past, present, future) are based on the number of probes.

Abbreviations: CSMFQ, Child Shortened Mood and Feelings Questionnaire; PANAS‐C, Positive and Negative Affect Schedule for Children; PANAS‐C‐P, Positive and Negative Affect Schedule for Children – Parent's version; PSMFQ, Parent Shortened Mood and Feelings Questionnaire; PSWQ‐C, Penn State Worry Questionnaire for Children; SCWBS, Stirling Children's Well‐being Scale.

The data on self‐reported mood and worry presented in Table 1 revealed that children, on average, exhibited high levels of positive mood and affect. This is reflected in their scores on the SCWBS and the positive affect subscale of the PANAS. Correspondingly, reports of negative mood and affect were relatively low, as indicated by the CSMFQ and the negative affect scale of the PANAS. By contrast, average levels of self‐reported worry fell closer to the midpoint of the PSWQ‐C. Compared to the more consistent reports of both positive and negative mood and affect, the distribution of scores on worry was more variable, indicating a broader range of worry experiences within the sample.

Carer reports reflect a similar trend. The shortened PANAS‐C‐P positive affect scores indicated that children experienced high levels of positive affect. Similarly, the shortened PANAS‐P negative affect scores and PSMFQ scores showed low levels of negative affect and low mood. However, when examining the correlations between the measures of children's self‐reported mood and the carers' reports of the children's mood, only one significant association emerged: between the SCWBS and the positive affect scale of the shortened PANAS‐C‐P (*ρ*(69) = 0.321, *p* = .007, BF_10_ = 8.15; see Table S2 in the Supplement for the full correlation matrix). This suggests a discrepancy between how children and their carers perceive the children's mood. Previous studies have also noted that children's self‐reports of mood can show weak relations with those reported by parents, particularly those of younger children (Edelbrock et al., 1986), and that parents can underestimate levels of low mood in their children (Angold et al., 1987). In this study, one possible source of lack of convergence in child and parent ratings of mood may be the difference in timing between the children's measures of mood and mind wandering (collected on the same day) and the parents' measures, which were completed over a longer period (spanning days before and after testing). This timing discrepancy may have introduced a confound, as parents' ratings could be based on a different reference period. For example, the instruction to consider “the past two weeks” could have meant a different timeframe for a participant completing their session on a Monday versus a parent completing the questionnaires on the Friday of the same week. Due to this procedural inconsistency and the lower response rates on carer‐reported measures (resulting from unreturned questionnaires), the parent‐reported data were excluded from further analysis. The analysis focused solely on children's self‐reported mood. Correlations between carer‐reported mood and mind wandering are presented in the Supplement (Table S3).

### Correlations between mind wandering and self‐reported measures of mood

Table 2 presents a correlation matrix examining the relations between mind wandering and self‐reported mood measures (for additional information see Table S4 in the Supplement). Added to the table is a composite variable for self‐reported negative mood. Its computation was informed by a recent meta‐analysis that scrutinized the relationship between well‐being and spontaneous thought, highlighting potential biases inherent in using single‐questionnaire measures of emotional affect, especially those with exclusively positive or negative wording (Kam et al., 2024). To create the composite variable for low mood (*M* = −0.11, *SD* = 14.89, skew = 0.68, kurtosis = 0.13) scores from the PANAS‐C negative affect and CSMFQ were standardized, while scores from the SCWBS and PANAS‐C positive affect were reverse‐scored and standardized. These standardized z‐scores were then summed to form the composite variable. Aggregating multiple indicators in this manner provides a more accurate measure of the underlying construct of negative mood (Song & Wang, 2012), enhancing the reliability and validity of statistical analyses by reducing measurement error and random variability associated with individual measures. The composite scale, consisting of 35 items, demonstrated strong internal consistency (Cronbach's α = .87).

**TABLE 2:** Zero‐order correlation matrix for age, self‐reported mood, and mind wandering.

	Mind wandering			
	Total	Future	Present	Past
Age				
*rho*	0.064	0.107	0.002	0.018
BF_10_	0.17	0.28	00.15	.15
*p*	.578	.355	.989	.878
SCWBS				
*rho*	−0.217	−0.135	−0.034	−0.221
BF_10_	1.66	0.36	0.16	2.09
*p*	.058	.240	.768	.053
SCWBS (social desirability score)				
*rho*	−0.043	−0.169	0.159	−0.133
BF_10_	0.16	0.75	0.73	0.39
*p*	.708	.142	.167	.248
CSMFQ				
*rho*	0.252	0.202	0.143	0.142
BF_10_	2.74	1.38	0.47	0.42
*p*	.**027**	.077	.214	.219
PANAS‐C negative affect				
*rho*	0.223	0.203	−0.022	0.294
BF_10_	1.79	1.47	0.15	14.50
*p*	.052	.076	.848	**.009**
PANAS‐C positive affect				
*rho*	−0.186	−0.055	−0.126	−0.180
BF_10_	0.68	0.18	0.34	0.92
*p*	.105	.633	.276	.118
PSWQ‐C				
*rho*	0.128	0.242	0.034	0.068
BF_10_	0.33	2.90	0.16	0.15
*p*	.266	.**034**	.870	.997
Negative mood composite				
*rho*	0.264	0.195	0.072	0.231
BF_10_	4.12	0.99	0.19	2.37
*p*	.**020**	.088	.532	.**043**

*Note*: Significant correlations highlighted in bold.

Abbreviations: BF_10_, Bayes Factor in favour of the alternative hypothesis; CSMFQ, Child Shortened Mood and Feelings Questionnaire; PANAS‐C, Positive and Negative Affect Schedule for Children; PSWQ‐C, Penn State Worry Questionnaire for Children; *rho*, Spearman's *rho*; SCWBS, Stirling Children's Well‐being Scale.

Spearman's correlations presented in Table 2 revealed a significant positive association between the overall rate of mind wandering and the CSMFQ (*ρ*(77) = .252, *p* = .027, BF_10_ = 2.74). Two additional measures, the SCWBS and the PANAS‐C negative affect scale, showed similar trends, though these were not statistically significant (*p* values of .058 and .052, respectively). Consistent with this overall pattern, the composite measure of negative mood was also positively correlated with the rate of mind wandering (*ρ*(77) = .265, *p* = .020, BF_10_ = 4.12). Age (*p* = .578) did not show a significant relationship with overall mind wandering, suggesting that the propensity for mind wandering remains relatively stable between the ages of 8 and 12.

Regarding the temporal orientation of mind wandering, the analysis revealed that future‐oriented mind wandering was positively associated with self‐reported worry as measured by the PSWQ‐C (*ρ*(77) = .242, *p* = .034, BF_10_ = 2.90). Total past‐oriented mind wandering was positively correlated with self‐reported negative affect as measured by the PANAS‐C negative affect scale (*ρ* = .294, *p* =. 009, BF_10_ = 14.50) the composite measure (*ρ*(77) = .231, *p* = .043, BF_10_ = 2.37). Mind wandering oriented towards the present did not show a significant relationship with any of the self‐reported mood measures. Additionally, age was not related to the frequency of mind wandering across any temporal orientation.

### Regression analyses

To test the hypotheses outlined in the Introduction, several generalized linear models were conducted. The predictors included in these analyses were selected based on the zero‐order correlations discussed in the previous section. The first model examined the relationship between self‐reported negative mood and overall mind wandering (Table 3). The likelihood ratio test indicates that the model containing the composite mood variable provides a significantly better fit than the null model (*Χ* ^2^ = 12.83, *p* < .001). Consistently, Bayesian Information Criterion (BIC) values also favour M1 over the null (ΔBIC = 8.66). In terms of parameter estimates, self‐reported negative mood emerged as a significant predictor of mind wandering, *z* = 3.58, *p* < .001, 95% CI [0.010, 0.036]. However, the overall model fit remained poor (Pearson goodness‐of‐fit test *p* = .008), suggesting that the model accounts for only a limited portion of the variance in mind wandering frequency. This highlights the likelihood that additional key determinants of mind wandering are not captured within the current model.

**TABLE 3:** GLMs examining mind wandering as the outcome variable.

	*B* (SE)	*z*	*p*	95% CI	
				Lower	Upper
Total mind wandering					
Intercept	−1.752 (0.745)	−2.352	.019	−3.215	−0.290
Age	0.005 (0.006)	0.720	.472	−0.008	0.017
Negative mood composite	0.023 (0.006)	3.582	**<.001**	0.010	0.036
Past mind wandering					
Intercept	−1.988 (1.219)	−1.632	.103	−4.373	0.425
Age	−0.005 (0.010)	−0.490	.624	−0.026	0.015
Negative mood composite	0.024 (0.010)	2.431	.**015**	0.004	0.044
Future mind wandering					
Intercept	−4.207 (1.158)	−3.632	<.001	−6.509	1.949
Age	0.011 (0.010)	1.186	.236	−0.008	0.030
PSWQ‐C	0.025 (0.020)	1.263	.207	−0.014	0.063

Abbreviations: B, unstandardised regression coefficient; B (SE), standard error for the unstandardised regression coefficient; *z*, standardized test statistic.

A second generalized linear model (Table 3) was fitted to examine whether increased negative mood predicts higher levels of past‐oriented mind wandering. Introducing the composite variable for negative self‐reported mood as an additional predictor significantly improved the model fit as indicated by the likelihood ratio test (*Χ* ^2^ = 5.70, *p* = .017) and marginally lower BIC value when compared to the null model (ΔBIC = 1.36). In the expanded model, negative mood emerged as a significant predictor of the frequency of past‐oriented thoughts (*z* = 2.43, *p* = .015, 95% CI [0.004, 0.044]). In this analysis, the Pearson goodness‐of‐fit statistic (*p* = .133) indicates a good fit.

Lastly, a generalized linear model analysis was conducted to determine whether self‐reported worry predicted rates of future‐oriented mind wandering (Table 3). As before, age was entered as a predictor in the null model. Adding the PSWQ‐C variable did not improve the model's fit (*X* ^2^ = 1.57, *p* = .210), and worry was not a significant predictor (*p* = .207).

## DISCUSSION

The present study aimed to enhance our understanding of the associations between mood and mind wandering in children, with a specific focus on the temporal orientation of mind wandering. The study had three main objectives: to explore associations between mood and the overall frequency of mind wandering, to investigate the link between lower mood and past‐oriented mind wandering, and to examine the relation between worry and future‐oriented mind wandering. The findings indicate that children reported engaging in mind wandering approximately 23% of the time, which is consistent with the estimates obtained in prior studies with children (i.e., Cherry et al., 2022, 2023; Keulers & Jonkman, 2019; Ye et al., 2014; Zhang et al., 2015). Lower mood was found to be associated with an increased propensity to mind wander. Generalized linear models, controlling for age, revealed that lower mood was a significant predictor of both the overall frequency of mind wandering and past‐oriented mind wandering specifically. Additionally, self‐reported worry was significantly related to levels of future‐oriented mind wandering, although this relation did not survive controlling for age. The implications of these findings are discussed in detail below.

### Low mood and the frequency of mind wandering

Our findings provide clear evidence that children who reported lower mood exhibited more frequent episodes of mind wandering, consistent with prior research conducted in both typical and clinical adult populations (Deng et al., 2012; Killingsworth & Gilbert, 2010; Poerio et al., 2013; Smallwood et al., 2009). The findings also align with previous research involving adolescents, which used self‐report measures to link mind wandering with lower mood (Mrazek et al., 2013) and depressive symptoms (Vannucci et al., 2020). Additionally, our results corroborate the findings of Jin et al. (2022) who used self‐report questionnaires to demonstrate a significant association between increased mind wandering and heightened negative emotions in children. This similarity in our findings to those of Jin et al. was obtained despite using very different measurement tools: probed reports of task‐unrelated thoughts, which are argued to offer higher fidelity than retrospective measures of mental events (e.g., Kane et al., 2021), and a composite measure of mood derived from different well‐established questionnaires (contrasting with the 7‐point scales targeting positive and negative emotions used by Jin et al.). Another point of extension is the age of the sample: while Jin et al.'s sample included children aged 9 to 14, we used a younger sample of 8–12‐year‐olds, meaning that it was restricted to middle childhood.

### Mental well‐being and the temporal orientation of mind wandering

Our results indicate that children who reported lower mood in our composite variable exhibited higher levels of past‐oriented mind wandering. This finding aligns with previous research in adult populations, which has consistently demonstrated an association between past‐oriented mind wandering and diminished emotional well‐being (Hoffmann et al., 2016; McKay et al., 2017; Poerio et al., 2013; Ruby et al., 2013; Smallwood et al., 2009). Additionally, our findings corroborate a recent study by Webb et al. (2022), which observed an association between negative affect and past‐oriented thoughts in adolescents aged 13–18 years. This link appears to be sensitive and robust enough to be observed even in typically developing children, highlighting the need for further investigation into the role of mind wandering in childhood.

We also found that an index of worry, operationalized through the scores obtained on the PSWQ‐C, was associated with future‐oriented mind wandering in our sample. To our knowledge, this is the first study to explore the relation between self‐reported worry and future‐oriented mind wandering in this age group. However, follow‐up generalized linear model analysis did not provide evidence for this relation. It is possible that a relation might be more in evidence if studied in a population of children with clinically significant anxiety who are known to have high levels of worry as measured using the PSWQ‐C (Păsărelu et al., 2016). We note also that we did not assess the valence of mind wandering episodes; it may be that the relation would have been easier to observe if we had singled out negatively valenced episodes of future‐oriented mind wandering.

We also observed similar rates of mind wandering across past, present, and future, suggesting that children in our sample did not display the prospective bias frequently observed in non‐clinical adult populations (e.g., Gilbert & Wilson, 2007; Smallwood et al., 2011; Song & Wang, 2012). While some studies have identified a prospective bias in children (Ye et al., 2014; Zhang et al., 2015), others have not (McCormack et al., 2018). Our findings align with McCormack et al.'s (2018) conclusion that increased future‐oriented thinking may develop later than middle childhood.

Our study's findings diverge from those of Jiang et al. (2021), who reported that thoughts about both the future and the past were associated with higher levels of well‐being in children and adolescents aged 11–18 years. This discrepancy may be partially attributed to the methodological differences between the studies; as we pointed out in the Introduction, Jiang et al.'s questionnaire method did not necessarily capture information about the frequency of past and future mind wandering per se. In contrast, our study employed thought probe methods to capture a state‐level measure of temporal mind wandering. The differences in our findings underscore the potential impact of measurement techniques on the observed relations between mind wandering and mood.

### Implications and future research directions

By establishing a link between mind wandering and mood in children, this study highlights the potential value of incorporating mind wandering assessments in non‐clinical educational settings. Identifying and targeting specific mind wandering patterns associated with poor well‐being in children could be helpful for developing effective early interventions. Mindfulness‐based programs, which have gained considerable attention, are often advocated for their potential benefits in improving mental health among young people and have also been widely suggested to reduce overall levels of mind wandering in adults (e.g., Feruglio et al., 2021; Mrazek et al., 2013). However, although there is certainly enthusiasm for mindfulness training, there is not yet a sufficiently firm evidence base supporting its effectiveness in young populations (e.g., Dunning et al., 2022). While mindfulness interventions have shown promise for certain outcomes, the overall quality of evidence with regard to children remains low and inconclusive.

Although our study adds new knowledge concerning the links between mood and mind wandering in children, we are aware that there are still many unanswered questions. We assessed mind wandering levels on a single occasion, providing only a snapshot of children's mind wandering. Additionally, testing was conducted via videoconferencing software and nearly half of the children reported their mind wandering and mood aloud in the presence of their parents (who were either beside them or in the same space), which may have led to social desirability effects. Addressing this limitation and incorporating ecological momentary assessment techniques over longer time periods could provide valuable insights by capturing real‐time data on the dynamic interplay between mood and mind wandering in natural settings. To further expand the research base, prospective studies could benefit from experimental designs, such as mood induction techniques, to investigate the causal relationships between mind wandering and mood in children. Furthermore, replicating studies like that of Poerio et al. (2013), which tracked the two‐way causal relations over time between adults' mind wandering and mood, could help identify whether similar patterns exist in children. Longitudinal studies would also be beneficial to understand how the relationship between mind wandering and mood evolves from childhood through adolescence, offering a more comprehensive view of this interaction over time.

## CONCLUSION

In conclusion, our research highlights a significant link between mind wandering and mood in children aged 8–12 years. We identified a clear association between low mood and the propensity to engage in mind wandering episodes. Additionally, our study revealed that low mood is a significant predictor of past‐oriented mind wandering and provided some, albeit weak, evidence of a relationship between worry and future‐oriented thought. These findings underscore the importance of further investigation into the dynamics between mood and mind wandering. Future research should employ experimental designs to elucidate the causal relationships between these variables. Understanding these dynamics in greater depth is crucial for developing targeted interventions aimed at improving emotional well‐being in children. By exploring how mood influences different types of mind wandering and vice versa, researchers can design more effective strategies to support children's mental health and enhance their overall emotional resilience.

## AUTHOR CONTRIBUTIONS

**Ellen Teague:** Conceptualization; data curation; writing – original draft; methodology; formal analysis; visualization. **Teresa McCormack:** Conceptualization; methodology; supervision; writing – review and editing. **Agnieszka J. Graham:** Data curation; supervision; formal analysis; visualization; conceptualization; methodology; writing – original draft; writing – review and editing.

## FUNDING INFORMATION

This research was conducted as part of the doctoral thesis of the first author and received no specific funding.

## CONFLICT OF INTEREST STATEMENT

The authors declare no conflict of interest.

## Supporting information


**Tables S1–S4**.

## Data Availability

The data that support the findings of this study are openly available at the Open Science Framework at https://osf.io/2a9ze/.
