# Supplementary material for: Low mood, worry and mind wandering in children
Source: Br J Dev Psychol. 2025 Mar 31;43(4):889–907. doi: 10.1111/bjdp.12561 (PMC12505825; doi:10.1111/bjdp.12561)
Supplement: Supplementary file 1 — Tables S1–S4. [file BJDP-43-889-s001.docx]

# Supplement **to ‘Low Mood, Worry, and Mind Wandering in Children’**

Abstract

This document presents supplementary material for the manuscript titled ‘Low Mood, Worry, and Mind Wandering in Children’.

*Keywords:* mind wandering, child development, mood, worry, temporal cognition

Contents

[Supplement to ‘Low Mood, Worry, and Mind Wandering in Children’ 1](#_Toc175551392)

[1. Measures of Mood and Affect 2](#_Toc175551393)

[The Stirling Children’s Wellbeing Scale 2](#_Toc175551394)

[The Positive and Negative Affect Schedule for Children 3](#_Toc175551395)

[Child Shortened Mood and Feelings Questionnaire 4](#_Toc175551396)

[Parent Shortened Mood and Feelings Questionnaire 5](#_Toc175551397)

[Positive and Negative Affect Schedule for Children - Parent’s version 6](#_Toc175551398)

[Penn State Worry Questionnaire: Child 8](#_Toc175551399)

[2. Temporal Orientation – Training Procedure 9](#_Toc175551400)

[3. Mind Wandering Task – Henry the Hedgehog Training Procedure 12](#_Toc175551401)

[4. Mind Wandering Task – Ollie the Owl Training Procedure 18](#_Toc175551402)

[5. Mind Wandering Task – Listening Activity Transcript 19](#_Toc175551403)

[6. Gender differences 24](#_Toc175551404)

[7. Correlations Between Self-Reported and Carer-Reported Measures of Mood 25](#_Toc175551405)

[8. Correlations Between Carer-Reported Mood and Mind Wandering 27](#_Toc175551406)

[9. Correlations Between Self-Reported Mood and Mind Wandering 29](#_Toc175551407)

# Measures of Mood and Affect

## The Stirling Children’s Wellbeing Scale


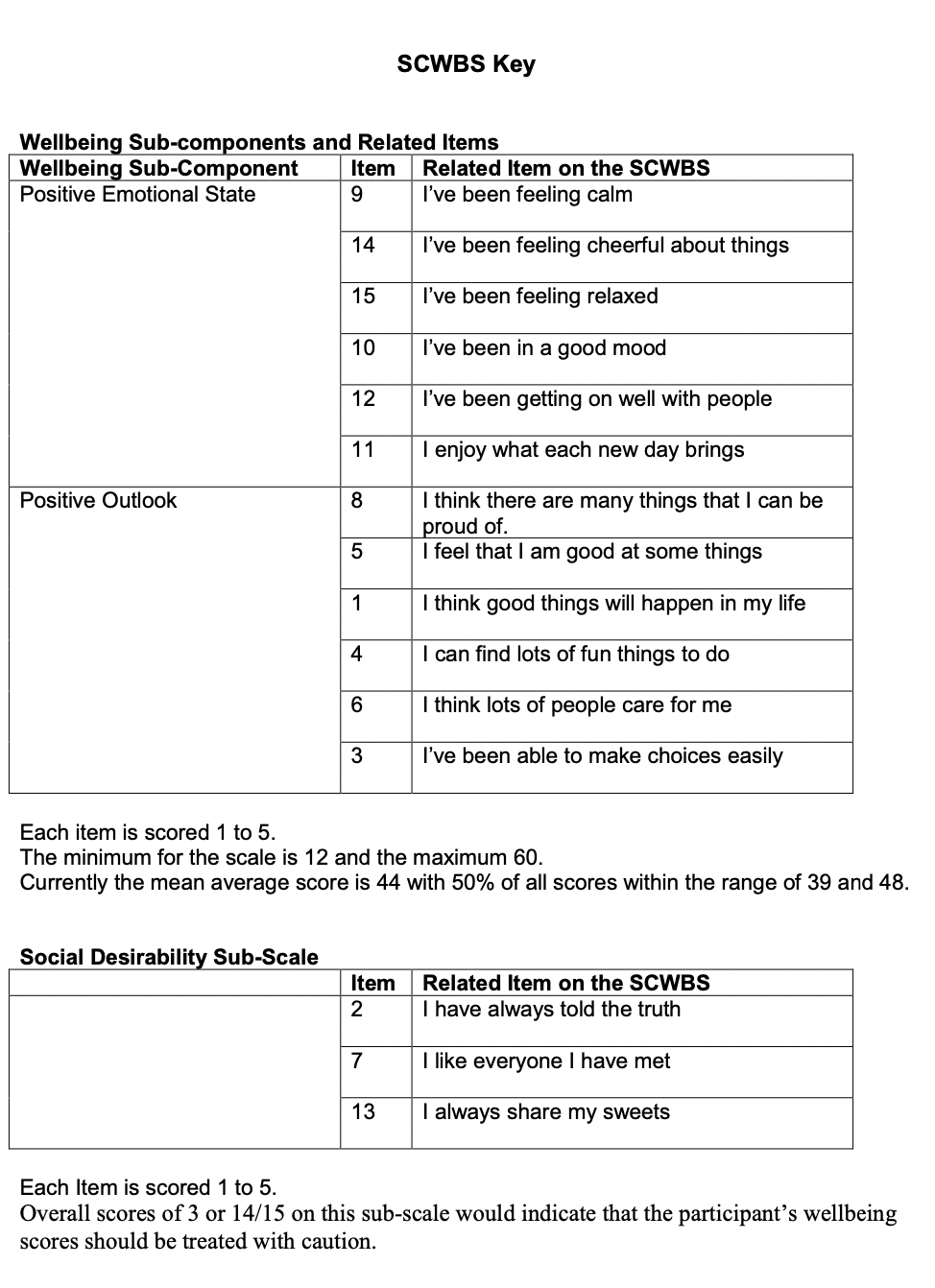

The SCWBS has shown good internal reliability with a Cronbach’s alpha of .85 (Liddle & Carter, 2015).

## The Positive and Negative Affect Schedule for Children

This scale consists of a number of words that describe different feelings and emotions.

Read each item and then circle the appropriate answer next to that word. Indicate how much you have felt this way during the past few weeks.

|  | Not much or not at all | A little | Some | Quite a bit | A lot |
| --- | --- | --- | --- | --- | --- |
| Joyful | 1 | 2 | 3 | 4 | 5 |
| Cheerful | 1 | 2 | 3 | 4 | 5 |
| Happy | 1 | 2 | 3 | 4 | 5 |
| Lively | 1 | 2 | 3 | 4 | 5 |
| Proud | 1 | 2 | 3 | 4 | 5 |
| Miserable | 1 | 2 | 3 | 4 | 5 |
| Mad | 1 | 2 | 3 | 4 | 5 |
| Afraid | 1 | 2 | 3 | 4 | 5 |
| Scared | 1 | 2 | 3 | 4 | 5 |
| Sad | 1 | 2 | 3 | 4 | 5 |

PANAS-C SCORING SHEET

Instructions: Record the child or adolescent’s rating for each item in the appropriate blank.

Ratings are from 1 to 5. To obtain the score for the positive affect (PA) scale, add the raw scores for the 5 items that compose the PA scale. Similarly, adding the raw scores for the 5 items that compose the negative affect (NA), scale the result in the score for the NA scale.

| Positive Affect (rated 1-5) _______ | Negative Affect (rated 1-5) _______ |
| --- | --- |
| Happy _______ | Sad _______ |
| Cheerful _______ | Mad _______ |
| Proud _______ | Scared ______ |
| Joyful _______ | Afraid _______ |
| Lively _______ | Miserable_______ |
|  |  |
|  |  |
| PA Total | NA Total |

The PANAS-C demonstrates strong internal reliability, with Cronbach’s alpha values of .86 and .82 for positive and negative items, respectively (Ebesutani et al., 2012).   

## Child Shortened Mood and Feelings Questionnaire
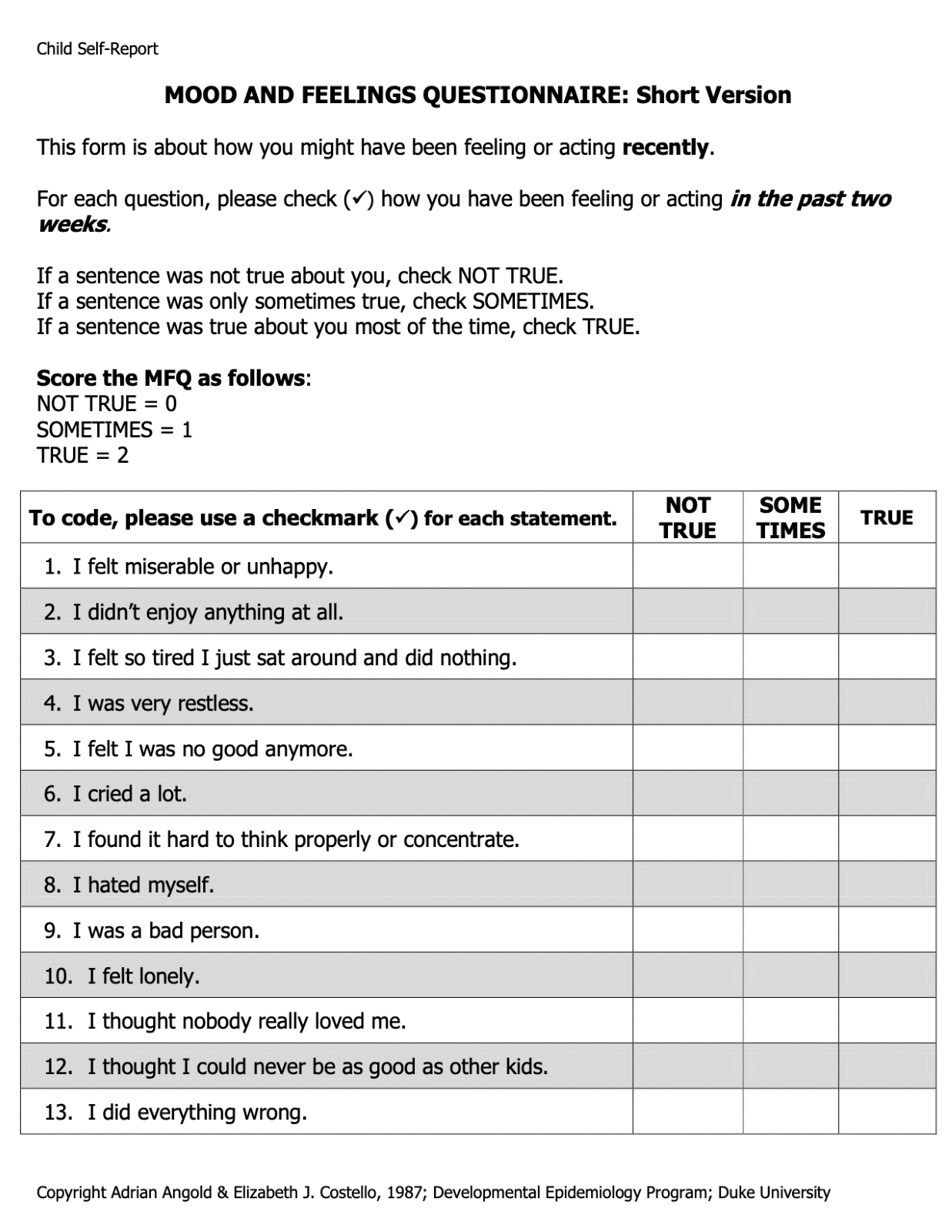


The CSMFQ demonstrates good internal reliability (Cronbach’s α ranging from .85 to .89; Jarbin et al., 2020; Kuo et al., 2005; Thabrew et al., 2018).

##
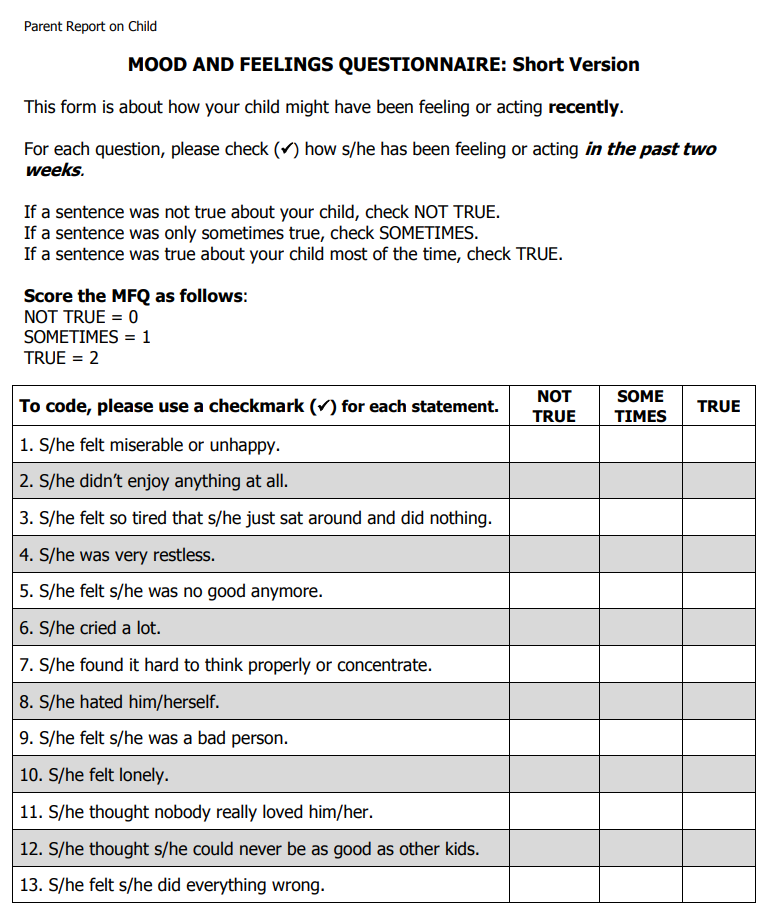
Parent Shortened Mood and Feelings Questionnaire

The PSMFQ demonstrates good internal reliability, with Cronbach’s alpha values between .85 and .89 (Jarbin et al., 2020).

## Positive and Negative Affect Schedule for Children - Parent’s version

This scale has a number of words that describe different feelings and emotions.

Read each item and then circle the best answer next to that word. Indicate to what extent your child has felt this way during the past few weeks. There are no right or wrong answers.

|  | Not much or not at all | A little | Some | Quite a bit | A lot |
| --- | --- | --- | --- | --- | --- |
| Interested | 1 | 2 | 3 | 4 | 5 |
| Sad | 1 | 2 | 3 | 4 | 5 |
| Frightened | 1 | 2 | 3 | 4 | 5 |
| Alert | 1 | 2 | 3 | 4 | 5 |
| Excited | 1 | 2 | 3 | 4 | 5 |
| Ashamed | 1 | 2 | 3 | 4 | 5 |
| Upset | 1 | 2 | 3 | 4 | 5 |
| Happy | 1 | 2 | 3 | 4 | 5 |
| Strong | 1 | 2 | 3 | 4 | 5 |
| Nervous | 1 | 2 | 3 | 4 | 5 |
| Guilty | 1 | 2 | 3 | 4 | 5 |
| Energetic | 1 | 2 | 3 | 4 | 5 |
| Scared | 1 | 2 | 3 | 4 | 5 |
| Calm | 1 | 2 | 3 | 4 | 5 |
| Miserable | 1 | 2 | 3 | 4 | 5 |
| Jittery | 1 | 2 | 3 | 4 | 5 |
| Cheerful | 1 | 2 | 3 | 4 | 5 |
| Active | 1 | 2 | 3 | 4 | 5 |
| Proud | 1 | 2 | 3 | 4 | 5 |
| Afraid | 1 | 2 | 3 | 4 | 5 |
| Joyful | 1 | 2 | 3 | 4 | 5 |
| Lonely | 1 | 2 | 3 | 4 | 5 |
| Mad | 1 | 2 | 3 | 4 | 5 |
| Fearless | 1 | 2 | 3 | 4 | 5 |
| Disgusted | 1 | 2 | 3 | 4 | 5 |
| Delighted | 1 | 2 | 3 | 4 | 5 |
| Blue | 1 | 2 | 3 | 4 | 5 |
| Daring | 1 | 2 | 3 | 4 | 5 |
| Gloomy | 1 | 2 | 3 | 4 | 5 |
| Lively | 1 | 2 | 3 | 4 | 5 |

Instructions: Record the parents rating for each item in the appropriate box

Ratings are from 1 to 5. To obtain the score for the positive affect (PA) scale, add the raw scores for the 12 items that compose the PA scale. Similarly, adding the raw scores for the 15 items that compose the negative affect (NA), scale results in the score for the NA scale.

| Positive Affect (rated 1-5) _______ | Negative Affect (rated 1-5) _______ |
| --- | --- |
| Interested _______ | Sad _______ |
| Excited _______ | Frightened _______ |
| Happy _______ | Ashamed _______ |
| Strong _______ | Upset _______ |
| Energetic _______ | Nervous _______ |
| Calm _______ | Guilty _______ |
| Cheerful _______ | Scared _______ |
| Active _______ | Miserable _______ |
| Proud _______ | Jittery _______ |
| Joyful _______ | Afraid _______ |
| Delighted _______ | Lonely _______ |
| Lively _______ | Mad _______ |
|  | Disgusted _______ |
|  | Blue _______ |
|  | Gloomy _______ |
| PA Total _______ | NA Total _______ |

 The shortened PANAS-C-P shows good internal reliability, with Cronbach’s alpha values of .85 for positive affect and .83 for negative affect (Ebesutani et al., 2011).

**Penn State Worry Questionnaire: Child**

**
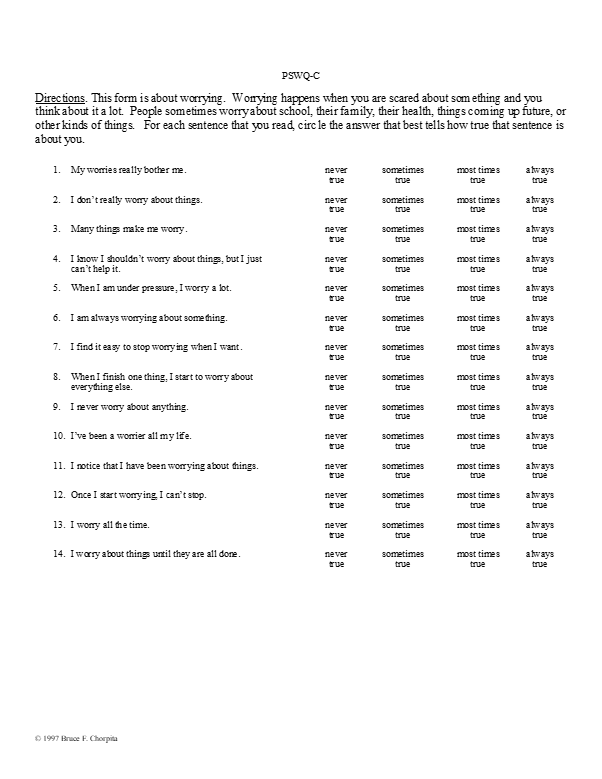
**

The PSWQ-C has good internal reliability (Cronbach’s α = .82; Muris et al., 2001).

# Temporal Orientation – Training Procedure

We are going to play a fun game; do you see these three squares? We are going to see if we can answer the questions by putting the pictures into the right squares.

Things that have happened already, such as what you had your breakfast this morning, go into this (purple) square that says the word Past.

Things that are happening right now, such as this game we are playing, go into this (green) square that says the word Now.

Things that haven’t happened yet, such as when what you might eat later today, go into this (orange) square that says the word Future.

**
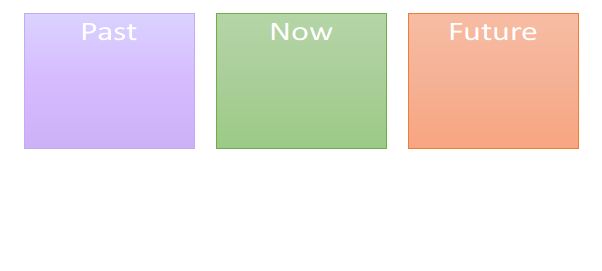
**

Check questions

“Which square do we put things into that *have already happened* like what you had for your breakfast this morning or a film you have already seen*?*” [Correct if wrong]

“Which square do we put things into that *are happening now* like this game we are playing?” [Correct if wrong]

“Which square do we put things into that *haven’t happened yet* like what you might eat later today or what you will watch on TV tonight?” [Correct if wrong]

**
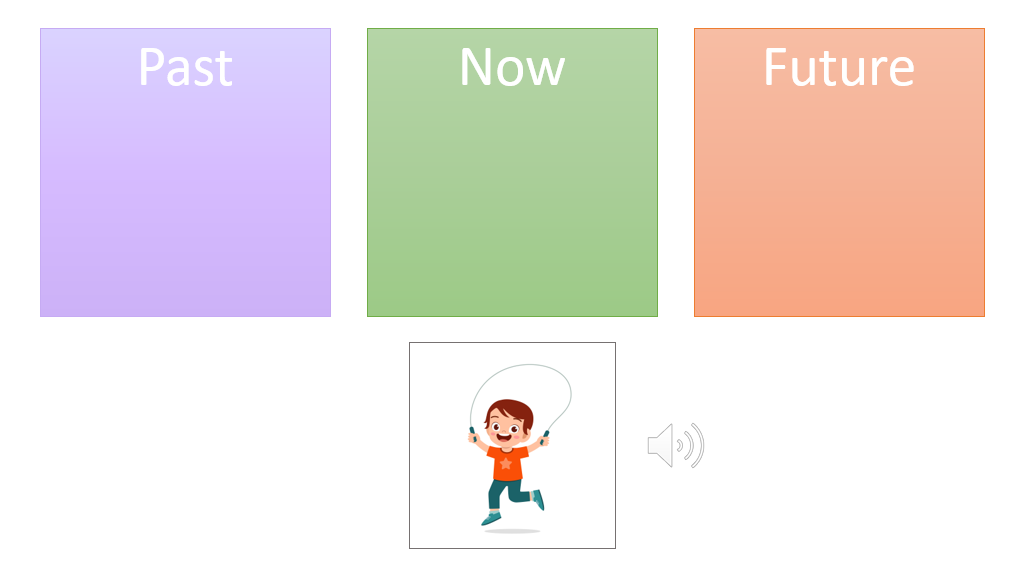
**

This is Timmy, and Timmy played with his skipping rope yesterday, so which square does this go in?

If correct: “That’s right, this is something that has already happened so that goes into this purple square here that says Past.”

If incorrect: “Remember, we put things that have already happened into this square. Tommy played with his skipping rope yesterday so that is something that has already happened, so we put it in this purple square here.”

[Picture will move to correct box on screen once the child has answered]

**
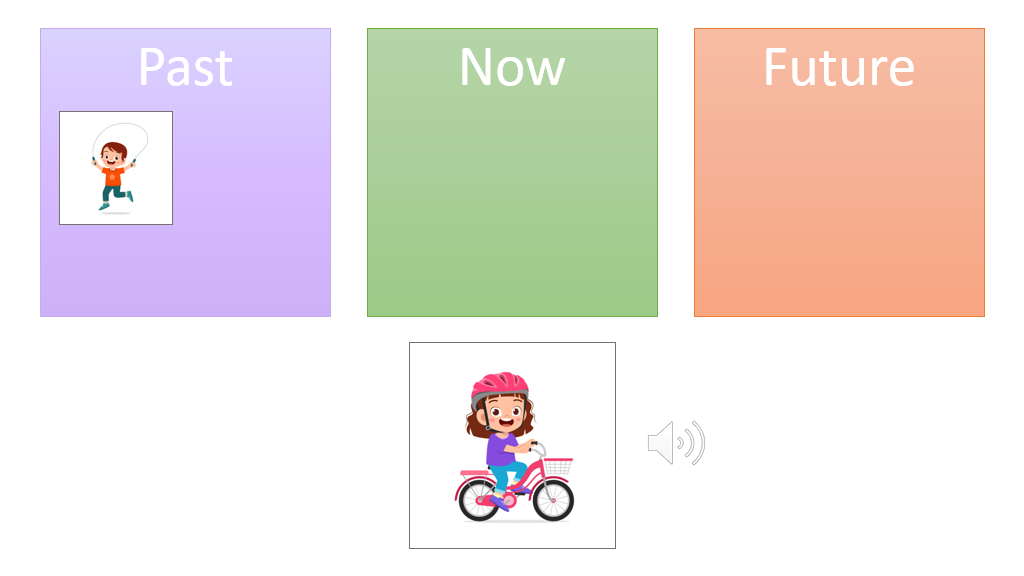
**

This is Susie, Susie is going to play on her bike tomorrow, so which square does this go in?

If correct: “That’s right, this is something that will happen so that goes into this orange square here.”

If incorrect: “Remember, we put things that haven’t happened yet into this square. Susie will play on her bike tomorrow so that is something that hasn’t happened yet, so we put it into this orange square here.”

**
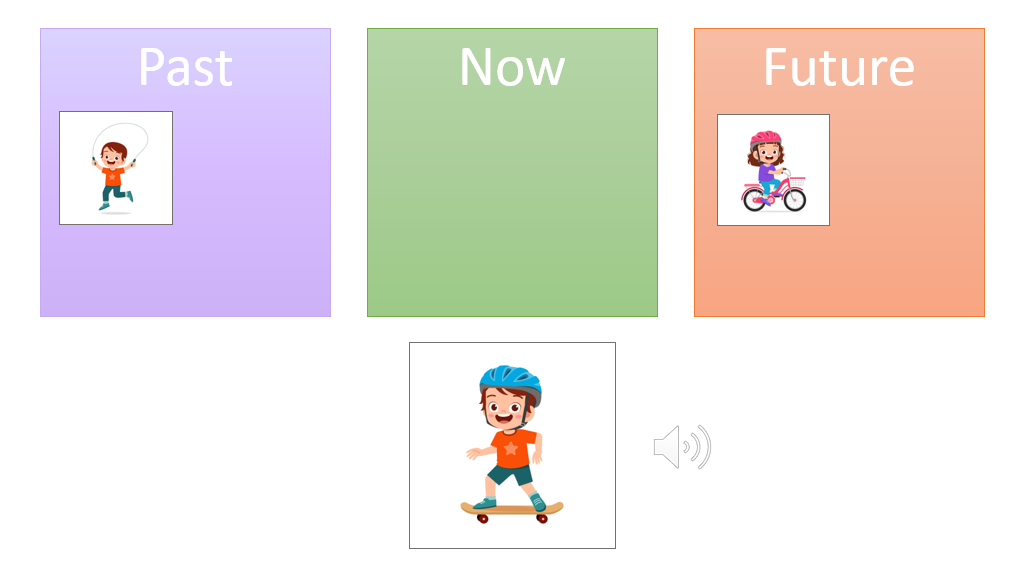
**

This is Timmy. Timmy is playing on his skateboard right now, so which box does this go into?

If correct, “That’s right. This is something that is happening now, so that goes into this green square here that says Now.”

If incorrect: “Remember, we put things that are happening now into this square. Timmy is playing on his skateboard right now so that is something that is happening in the here and now, so we put it in this green square’.

 Further examples:

- Past event – Susie walked her dog yesterday
- Future event – Tommy will visit his grandparents tomorrow

# Mind Wandering Task – Henry the Hedgehog Training Procedure

**
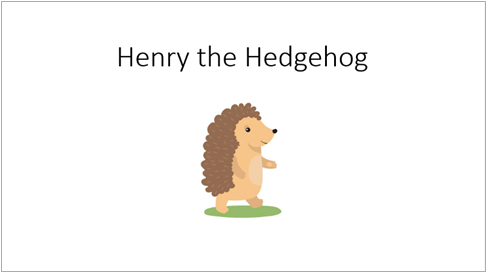
**

“This is Henry the hedgehog. Henry loves reading books.”

**
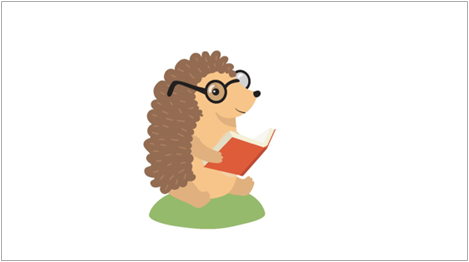
**

“You can see here that Henry is busy reading one of his favourite books, a book about sunflowers. Henry is trying really hard to pay attention to the book, but always paying attention is impossible. Often Henry might start to think of something other than the story. Let’s have a look at some of Henry’s thoughts.”

**
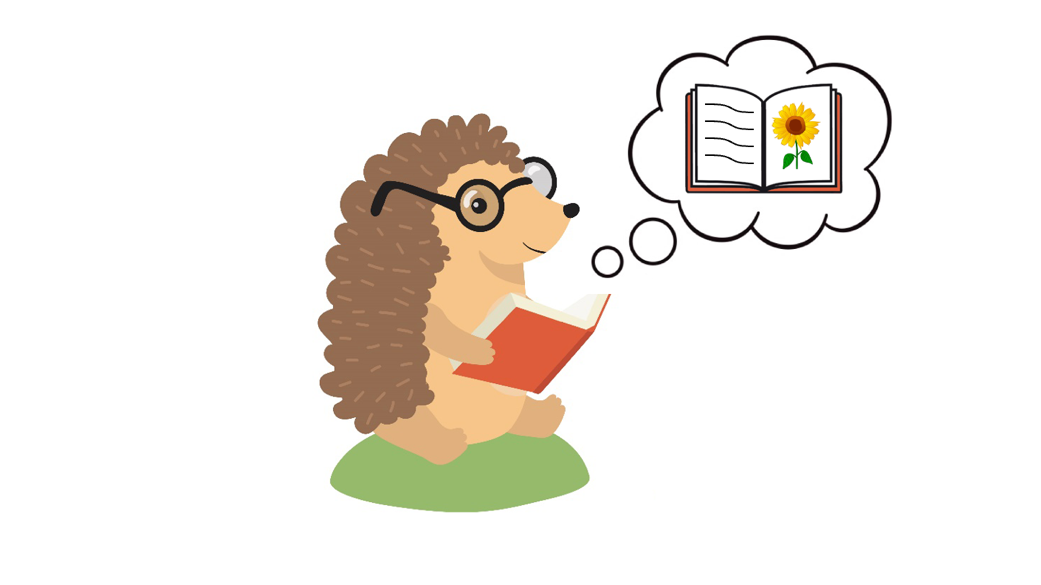
**

“Here Henry is thinking about the story about sunflowers and nothing else. His thoughts match what he is doing, and no other thoughts have popped into his head. Henry is just thinking about the story he’s reading.”

**
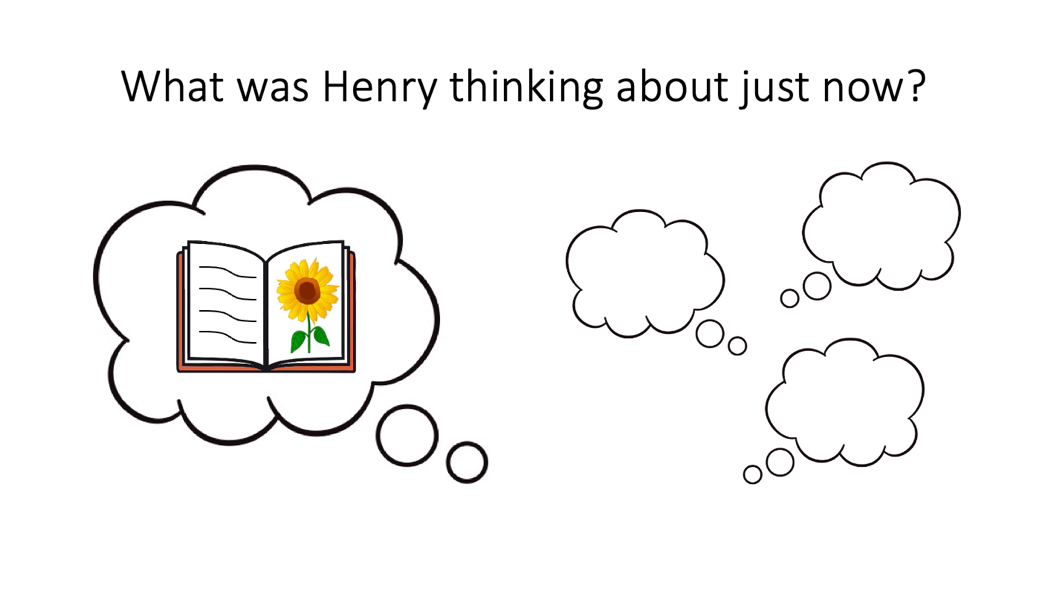
**

Here we have a thought bubble with a picture of Henry’s story in it, this is for when Henry is thinking about his story, and on the other side there are lots of different thought bubbles – these are for when Henry is thinking about something else, not his story.

“Now, what was Henry thinking about just now? What should we pick? Was Henry thinking about the story or something else?

*[Wait for child’s response]*

Yes/no, Henry was thinking about the story he is reading – so we have to pick this bubble which has a picture of Henry’s story book inside it.

*[If the participant gets a question wrong the researcher will say “Not quite, let’s go over that again” and repeat the relevant information (e.g., “Henry was thinking about the sunflowers, and nothing else…”). The participant will then have a second opportunity to answer the question correctly. If this second attempt is unsuccessful, the research will provide the correct answer and move on to the next section of the training exercise. If the participant answers two or more questions incorrectly, the training will be repeated using a different story (featuring a different character) which follows the same basic structure.]*

**
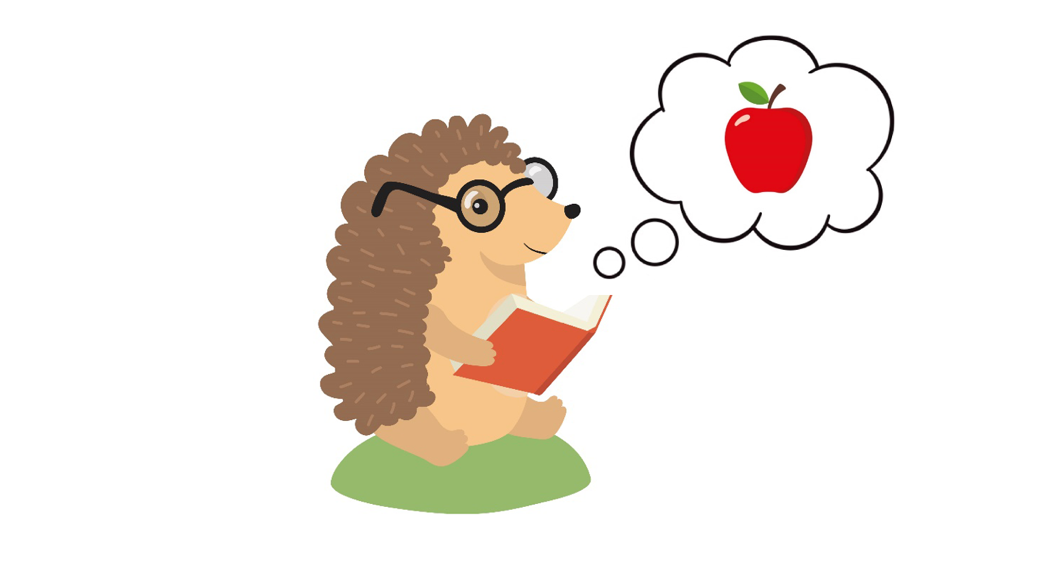
**

Let’s take another look at Henry’s thoughts.”

“While Henry is reading, he starts to feel hungry and now he is thinking about what he would love to eat for lunch later. You can see here that Henry is thinking of eating a lovely red apple, in fact he is not even paying attention to the words in his book, so he gets a bit lost and has to go back and read the page again.”

PROBE: “What was Henry thinking about just now? Which bubble should we pick? Was Henry thinking about the story or something else?

*[Wait for child’s response]*

Yes, Henry was thinking about something else – he was thinking about the red apple that he would like to eat for lunch later. So, we have to pick these other thought bubbles.

**
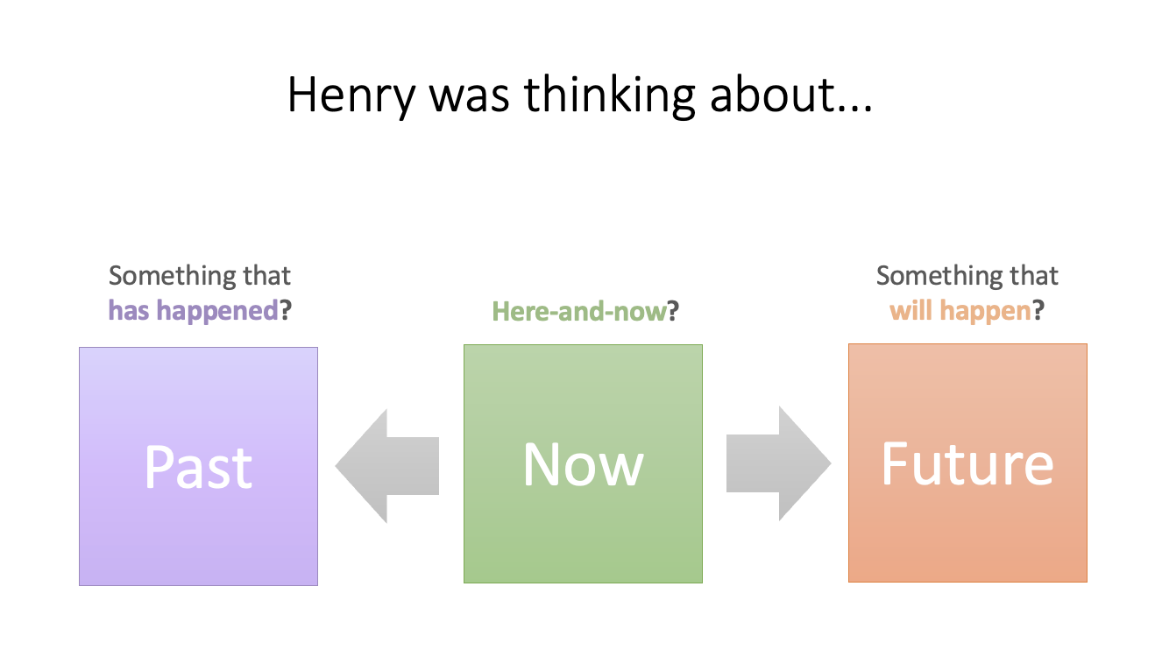
**

“Now this time, because we said Henry was thinking about something else, not his story, we get to answer another question. Remember that Henry was thinking about what he would like to each for lunch later that day. So, was the thing that Henry was thinking about just now something that has already happened, something that is happening right now, or something that will happen later? Which arrow should we pick? The purple box that says past - for something that has already happened, the green box that says now, for something that is happening right now, or the orange box which says future - for something that will happen?”

*[Wait for child’s response]*

“Yes/no, Henry was thinking about something that will happen, so we need to pick the orange box that says the future – so something that will happen.”

**
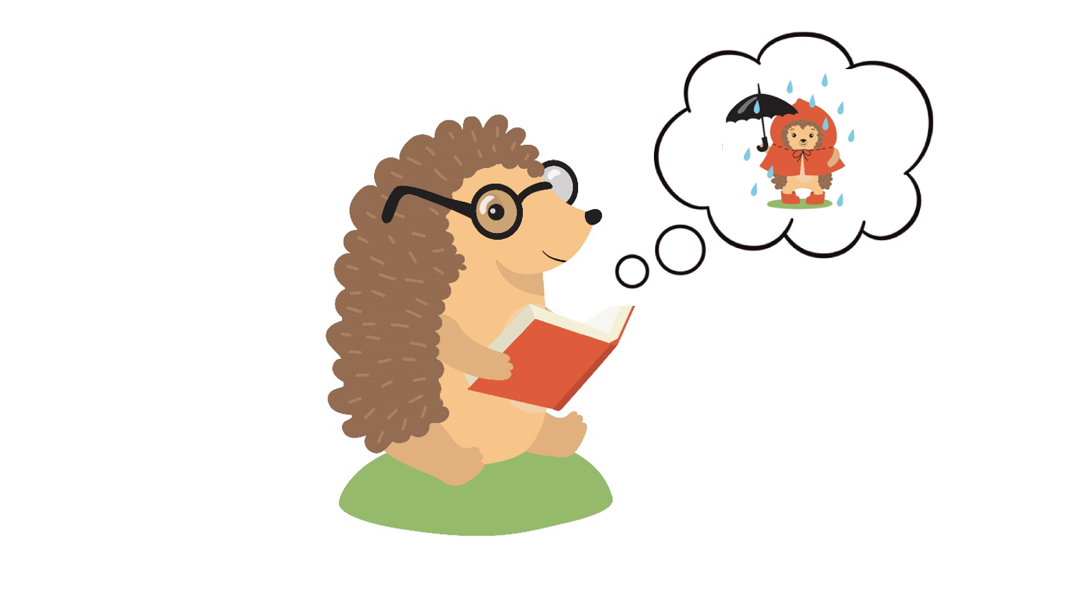
**

“Henry goes back to reading his book, and then he begins thinking about things that happened earlier that day. Earlier on Henry went out to play with his friends, but it started to rain, and Henry got soaked and had to come back home.

PROBE: “What was Henry thinking about just now? What should we pick? Was Henry thinking about the story or something else?

*[Wait for child’s response]*

Yes, Henry was thinking about something else – he was thinking about being out in the rain earlier that day. So, we have to pick these thought bubbles for something else.

**
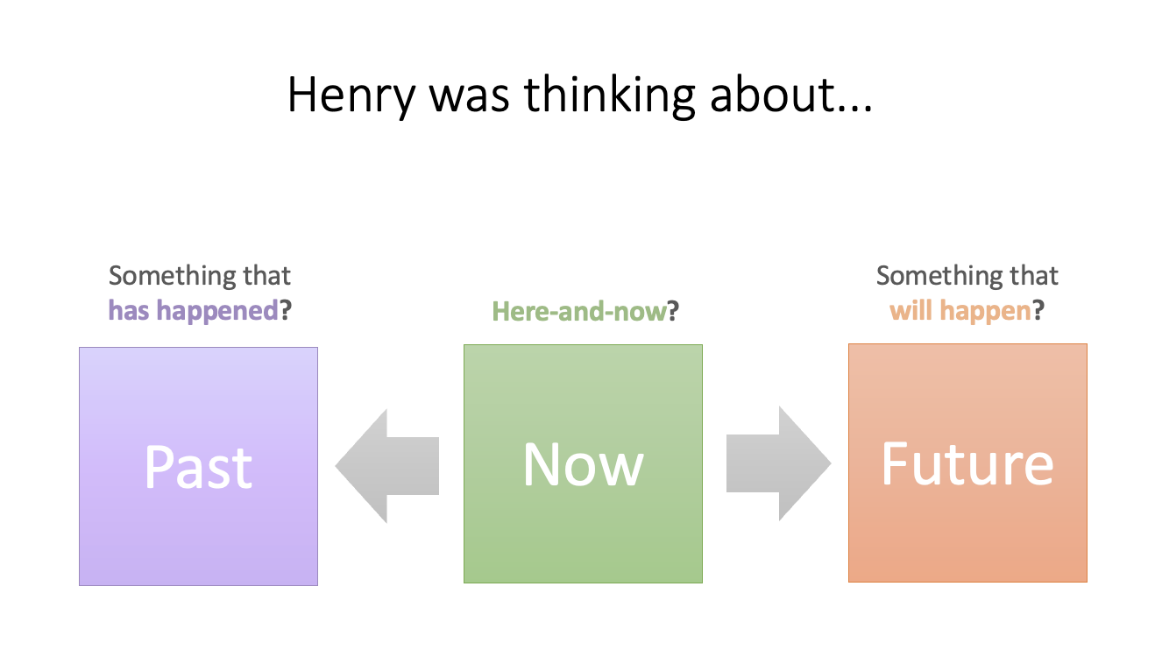
**

“Remember that Henry was thinking about wanting to play with his friends and being out in the rain earlier that day. So, was Henry thinking about something that has already happened, something that is happening right now, or something that will happen later?

*[Wait for child’s response]*

Yes/no, Henry was thinking about his walk he went on earlier that day, so we pick the purple box that says past– so, something that has happened.”

“Let’s take another look at Henry’s thoughts. He starts reading the story about the sunflowers again.”

**
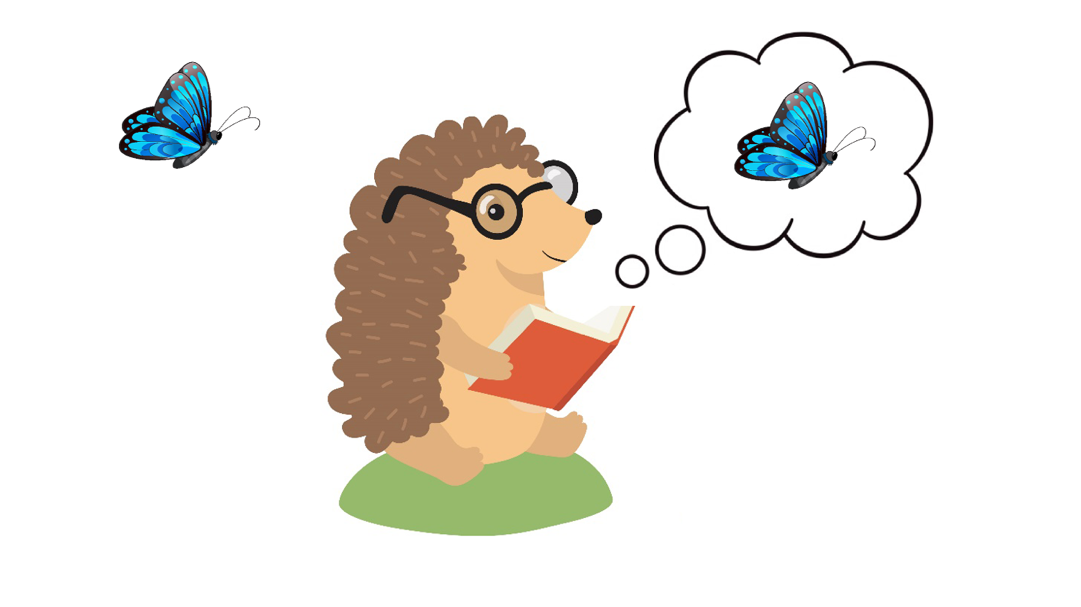
**

“Henry is busy reading his book when, all of a sudden, a blue butterfly appears in his room and flies around Henry’s head.”

“Henry is now thinking about the butterfly and he’s not paying attention to his book anymore.”

PROBE: “What was Henry thinking about just now? What should we pick? Was Henry thinking about the story or something else?

*[Wait for child’s response]*

**
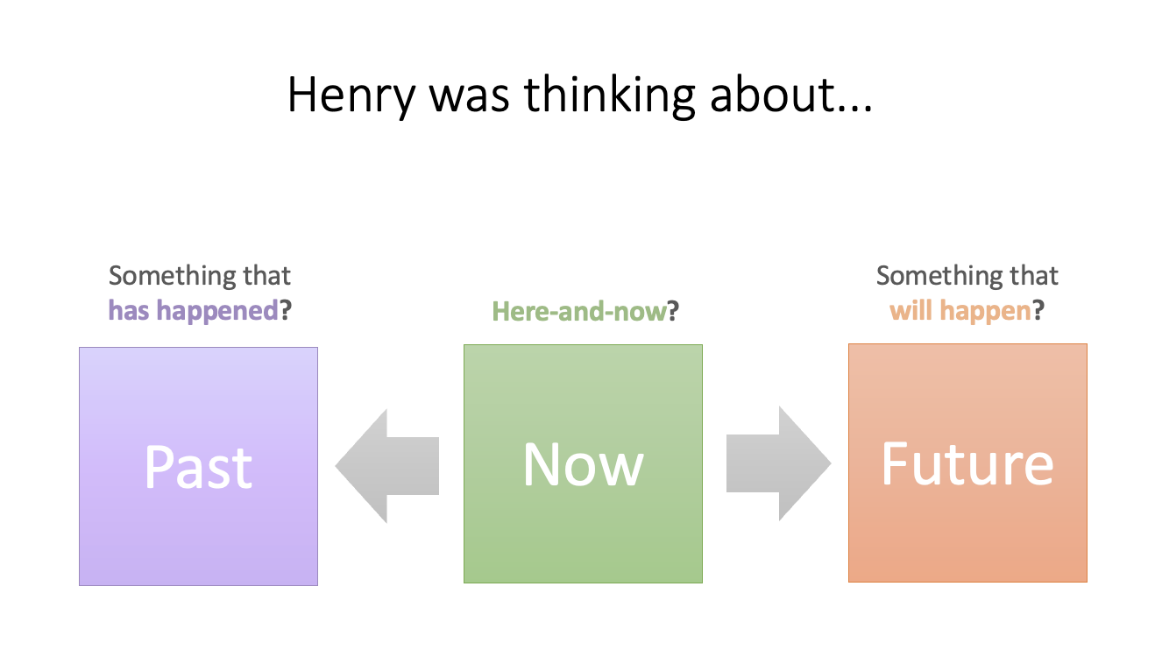
**

Yes, Henry was thinking about something else – he was thinking about the blue butterfly flying around. So, we have to pick these thought bubbles for something else.

“Remember that Henry was thinking about the butterfly flying around. So, was Henry thinking about something that has already happened, something that is happening right now, or something that will happen later?

*[Wait for child’s response]*

Yes/no, Henry was thinking about the butterfly, so we pick the green box that says Now – so something that is happening now.”

# Mind Wandering Task – Ollie the Owl Training Procedure

The second training procedure will follow the same script as the one outlined in detail above.


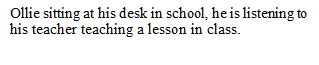
**
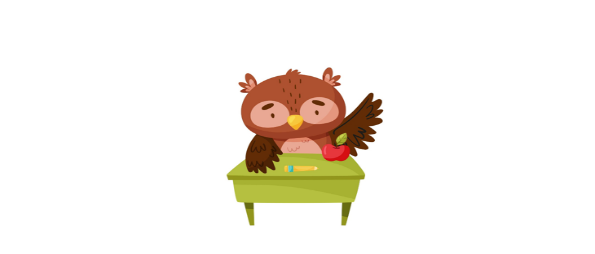
**

**
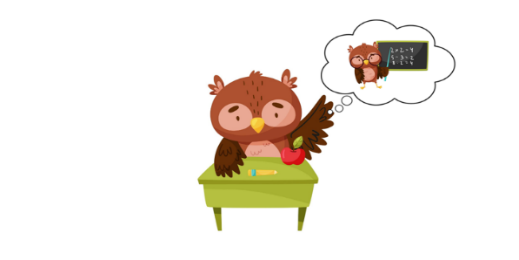

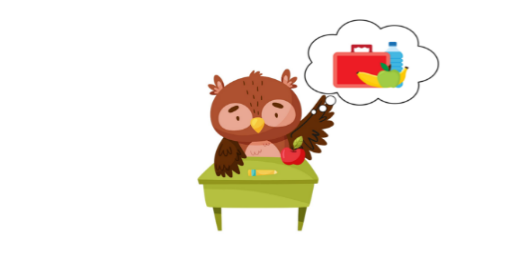
**

**
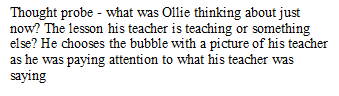

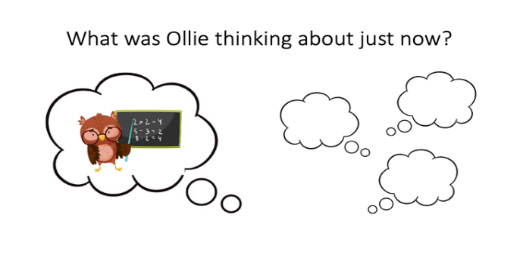

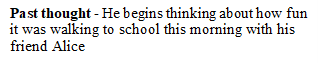

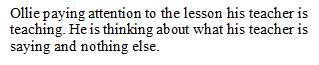

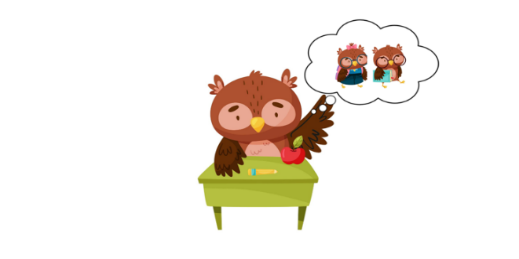
**

**
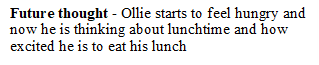
**

**
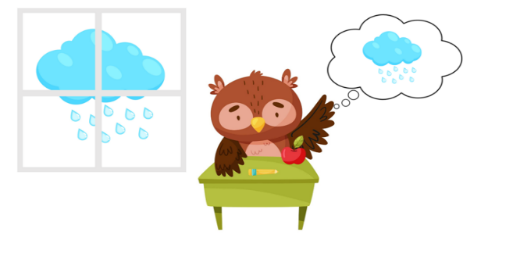
**

**
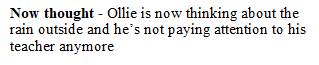
**

# Mind Wandering Task – Listening Activity Transcript

Are you ready to begin the story?

__________________________________________________________________________

Today, I am going to tell you a story about an Egyptian Pharaoh from a very long time ago. This Pharaoh was called Olufemi, during this story I will refer to them as Pharaoh Olu for short.

You have maybe heard about ancient Egypt; it was one of the first civilisations in the world and people today are still fascinated by it.  A civilisation is a group of people who share things like language, laws and a certain way of life. You are living in a civilisation right this second!

Anyway, back to ancient Egypt, it was one of the first civilisations we know about!

So, where was ancient Egypt? Ancient Egypt and modern-day Egypt is located in the northeastern part of Africa. Africa is one of the world’s seven continents. A continent is basically a large solid area of land and there can be many countries within a continent. So, Egypt is one of the countries which is located in the continent of Africa.

Ancient civilisations lived near rivers this was because of the water.

These people, like us, needed the water to drink and to water their crops. Crops are grown to keep us alive. There are different kind of crops, but for an ancient civilisation food crops would have probably been the most important. So early civilisations needed rivers for water and food.

***Thought probe (example below).***Note that all other probes will follow the same structure.

**
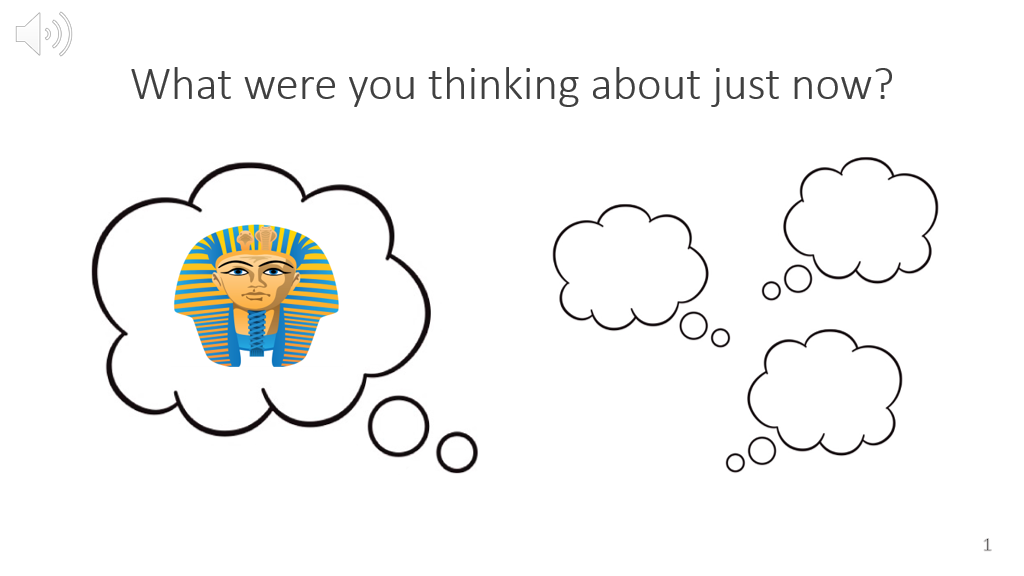
**

What were you thinking about just now? What was just said in the story or something else? Remember there are no right or wrong answers.

*(if ‘Something else’)*

**
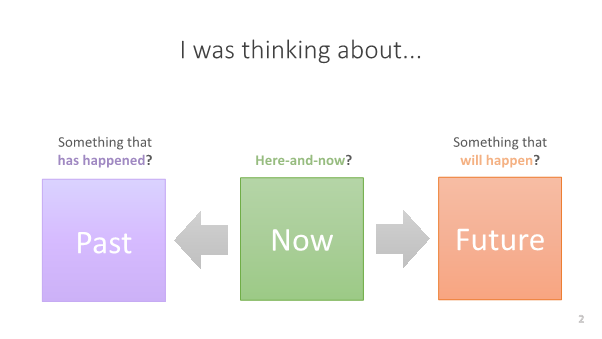
**

*(if ‘The Story’)*

**
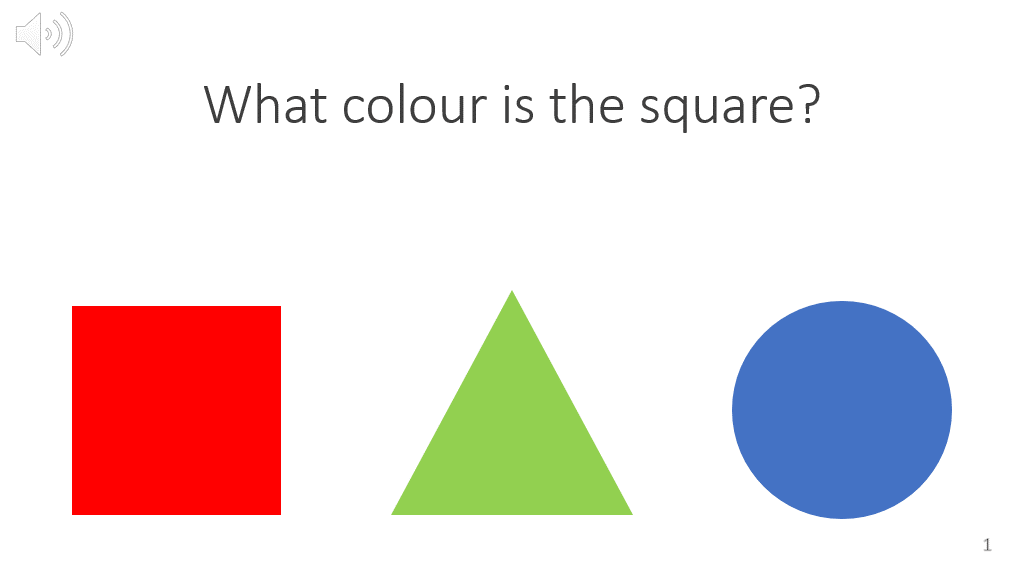
**

What colour is the square?

**Listening Activity Transcript continued**

The river that was near the civilisation of Ancient Egypt was the river Nile. The river Nile still runs through Africa today; it is actually the longest river in the world.  The same river that helped the people of ancient Egypt is still helping people today. The river Nile really helped make ancient Egypt such a great early civilisation.

The Egyptian pyramids are well known; they are ancient pyramid-shaped structures located in Egypt. The pyramids are large structures with four sides that slope upwards to meet at a point, the point is called the apex. The sides are made up of triangles but the pyramids in Egypt are actually known as square pyramids this is because their bases are square. It is amazing that they were built a very long time ago and they are still standing today! Most of the Egyptian pyramids were built as tombs for the country’s important people during ancient times. What that means is that in ancient Egypt, when someone important had died their body would be placed in a tomb underneath the pyramids.

Ancient Egyptians were kept busy by building pyramids! Have you ever heard of an archaeologist? An archaeologist is a person who looks at very old objects and places to learn things about the past. Archaeologists have found over 130 pyramids in Egypt and they are still looking for more! The pyramids would get built very, very slowly.

***Thought probe***

The Great Pyramid of Giza took 23 years to build but that is hardly surprising when you think about the weight of those blocks of rock and how high the pyramid is. It is believed that it took about 20,000 workers to build it. Amazing. I will come back to this later, but the Great Pyramid of Olu also took a long time to build, about 19 years!

There are some theories as to how the pyramids were built, but no-one knows for sure.

The Ancient Egyptians were great at inventing and invented a lot of the things we still use today!  They invented medicine, musical instruments, paper, pens, locks, keys, make up and even… toothpaste!

People had many different jobs in ancient Egypt, there were farmers, craftspeople, soldiers, and priests and priestesses. There were also people called scribes, who were very important in running the country and they were considered to be very powerful. Why? Well, they were the only people who could read and write, and they helped run the country.

Pharaohs in ancient Egypt wore ‘false beards’, this accessory was very important for making a Pharaoh look very powerful. Most false beards were made from metal, such as copper, gold, silver and attached to the Pharaoh’s chin. Pharaoh Olu’s false beard was made out of lead. Both men and women Pharaohs wore false beards.

***Thought probe***

So, I’m going to tell you about a Pharaoh in ancient Egypt. The Pharaoh was the king or queen of everything in ancient Egypt. The people believed the Pharaoh was a child of the gods and after death he also became a god.

You have perhaps not heard much about Pharaoh Olu before; Pharaoh Olu was born a very long time ago in the year 900BC. Olu became an Egyptian pharaoh at 12 years old, making Olu one of the youngest Pharaoh’s ever! We didn’t know about Olu for a very long time, the tomb under the pyramid dedicated to Pharaoh Olu was left undiscovered for over 3000 years, it wasn’t until very recently that people finally found the tomb of Pharaoh Olu!

But let us go back to when Pharaoh Olu was alive! Olu had just become the Pharaoh of ancient Egypt. When someone became a Pharaoh in ancient Egypt, they had to wear big headdresses and lots of jewellery. Olu liked to wear lots of jewellery but most of all Olu loved big and colourful headdresses. Pharaoh Olu’s favourite one was a dazzling gold with vibrant blue stripes! This matched Olu’s false beard which was decorated with blue spots. Pharaoh Olu also liked to wear red robes; the fabric of Olu’s robes was made from crinkle cotton. This fabric is great for helping people stay cool, this was important as the sun is very hot in Egypt!

***Thought probe***

On very hot days in Egypt the temperature can reach 48 degrees Celsius. That is very hot! This is why it was so important for Pharaoh Olu to wear clothes that helped to keep their body feel cool. As it was so hot during the day, Pharaoh Olu also liked to eat refreshing snacks to keep cool. Pharaoh Olu liked to eat moon drop grapes. Grapes are mostly made up out of water, so they make a very refreshing snack. So, grapes helped to keep Pharaoh Olu from feeling too thirsty on the sunny days in ancient Egypt.

The civilisation of ancient Egypt lasted for many, many years and because of this there were at least 170 different pharaohs who ruled in ancient Egypt at different times. For this reason, when Olu become a Pharaoh, Olu decided they wanted to leave behind a big legacy. What this means is that Pharaoh Olu really wanted people to remember their rule forever and ever. So, Olu thought a good way to do this would be to build a very big statue next to the pyramids. Olu sat on a fancy golden throne made just for the Pharaoh of ancient Egypt, Olu was being fanned with Olu’s favourite ostrich feather fan while Olu pondered what kind of statue they could build.

***Thought probe***

Pharaoh Olu was discussing the statue they wanted to build with a very important priestess of the time, she was called Neith. Pharaoh Olu and priestess Neith finally came to a decision; they decided the statue had to be big – gigantic and strong! This was so the statue could stand the test of time and people could come to see the statue for years and years to come.

Pharaoh Olu decided to get the workers of ancient Egypt to build the Bonx. The Bonx is a funny kind of statue because it has the body of a lion but the head of buffalo! This statue was very valuable to Pharaoh Olu and all the people of ancient Egypt. I will come back to this later but lots of different kinds of animals were very important to ancient Egyptian culture. 
So, Pharaoh Olu built the Bonx and the statue is thousands of years old!  The statue is very, very old and it is actually now missing its nose! Oh no! How did this happen? Hm, well some people think it maybe got damaged a long time ago, maybe from a cannonball hit after being used for target practice. But I can tell you those people are wrong. I know what really happened to the Bonx’s nose! One very sunny day in Egypt, the Bonx was sunbathing when the statue felt a tickle in its nose…AA-CHOO. It was then that the Bonx sneezed a little too hard and the statue’s nose fell off!

***Thought probe***

Oh no, the Bonx really misses its nose, and one thing is for certain, the Bonx would really like its nose back. So, if you happen to find the Bonx’s nose please do return it to the statue and bring some superglue to help him stick it back on! Once Pharaoh Olu’s workers had finished building the Bonx, Olu wondered what else to do to help build his legacy? Pharaoh Olu really wanted to make sure people would remember them. Pharaoh Olu discussed the matter with an important Egyptian priest of the time, the priest was called Zosar. Pharaoh Olu and Zosar decided they would need to have the workers build a very impressive pyramid with a very magnificent tomb underneath that pyramid. Pharaoh Olu hired a lot of workers to build this pyramid. They decided to name the pyramid to be named ‘the Great Pyramid of Olufemi’. Underneath this pyramid, Pharaoh Olu decided to transform an underground cave into a cosy tomb. Pharaoh Olu had several requirements for this tomb, Olu wanted the tomb to be within a group of very important pyramids called ‘Valley of Pharaohs’. Pharaoh Olu wanted a very big tomb so that Olu’s golden throne and all Olu’s relics could fit inside it. Pharaoh Olu wanted this tomb to be very comfortable, and it is just as well because Olu would be left undisturbed until it was discovered in the year 2018. That means Pharaoh Olu was left alone in the tomb for over 3000 years, that is a very long time!

***Thought probe***

Sadly, Pharaoh Olu did die one day, after eating one too many prickly cucumbers, Olu was placed in this fancy tomb. When an important person had died in ancient Egypt the people would throw a big ceremony for that person. Lots of priests and priestesses would help to place the important person in their tomb. The very powerful priests and priestesses in ancient Egypt and liked to wear liked to wear purple robes and slippers.

Before the story ends, let’s talk about cats! Cats are one of the most popular pets in the world, cats have been kept as pets for thousands of years. There are lots of different types of cats, they can be different colours and sizes. Cats can have long hair, short hair and some have no hair at all! The people of Ancient Egypt believed cats to be sacred and holy. What this means is that in Ancient Egypt the people practically worshipped cats! Pharaoh Olu was no exception, when Olu was alive, Olu had two very special cats, they were called Fluffy and Sooty. They were both Siamese cats with striking orange eyes and cute little noses that were as black as coal. Pharaoh Olu loved these cats very much and treated them very well. They both had collars with bells, this meant Pharaoh Olu could always hear them coming as their bells made tinkling noises when they walked.

***Thought probe***

Well now our story is coming to an end and over 3000 years have passed since the events in this story took place. Now, Pharaoh Olu’s tomb has now been discovered, along with all the ancient artefacts. They have all been moved to a museum. So, if you’re ever visiting Egypt please make sure to pop into the museum and say hi to all the pharaohs.

# Gender differences

**Table S1**

*Gender Differences across All Measures of Interest*

|  | *U* | *p* | BF_10_ |
| --- | --- | --- | --- |
| Age | 639.00 | .445 | 0.28 |
| SCWBS | 751.50 | .692 | 0.33 |
| SCWBS social desirability score | 1047.00 | **<.001** | 100.91 |
| CSMFQ | 602.50 | .250 | 0.43 |
| PANAS-C negative affect | 545.00 | .080 | 0.83 |
| PANAS-C positive affect | 802.50 | .353 | 0.37 |
| PSWQ-C | 773.00 | .536 | 0.29 |
| PANAS-C-P negative affect | 605.50 | .640 | 0.26 |
| PANAS-C-P positive affect | 505.00 | .609 | 0.28 |
| PSMFQ | 593.50 | .747 | 0.31 |
| Total mind wandering | 755.50 | .656 | 0.25 |
| Future mind wandering | 661.50 | .556 | 0.32 |
| Present mind wandering | 869.50 | .072 | 0.89 |
| Past mind wandering | 657.50 | .512 | 0.26 |
| Negative mood composite | 601.50 | .249 | 0.57 |

*Note.* Significant differences highlighted in bold. U = Mann-Whitney U. BF_10_ = Bayes Factor in favour of the alternative hypothesis. SCWBS = Stirling Children’s Wellbeing Scale; CSMFQ = Child Shortened Mood and Feelings Questionnaire; PANAS-C = Positive and Negative Affect Schedule for Children; PSWQ-C = Penn State Worry Questionnaire for Children; PANAS-C-P = Positive and Negative Affect Schedule for Children – Parent’s version; PSMFQ = Parent Shortened Mood and Feelings Questionnaire.

# Correlations Between Self-Reported and Carer-Reported Measures of Mood

**Table S2**

*Zero-Order Correlations for Self-Reported and Carer-Reported Measures of Mood and Affect*

|  |  | Age | SCWBS | SCWBS (social desirability score) | CSMFQ | PANAS-C negative affect | PANAS-C  positive affect | PSWQ-C | PANAS-C-P negative affect | PANAS-C-P positive affect | PSMFQ |
| --- | --- | --- | --- | --- | --- | --- | --- | --- | --- | --- | --- |
| Age | *rho* | — |  |  |  |  |  |  |  |  |  |
|  | BF_10_ | — |  |  |  |  |  |  |  |  |  |
|  | *P* | — |  |  |  |  |  |  |  |  |  |
| SCWBS | *rho* | -.125 | — |  |  |  |  |  |  |  |  |
|  | BF_10_ | 0.30 | — |  |  |  |  |  |  |  |  |
|  | *p* | .280 | — |  |  |  |  |  |  |  |  |
| SCWBS (social desirability score) | *rho* | -.114 | .389 | — |  |  |  |  |  |  |  |
|  | BF_10_ | 0.26 | > 100 | — |  |  |  |  |  |  |  |
|  | *p* | .324 | **< .001** | — |  |  |  |  |  |  |  |
| CSMFQ | *rho* | .074 | -.445 | -.048 | — |  |  |  |  |  |  |
|  | BF_10_ | 0.19 | > 1000 | 0.17 | — |  |  |  |  |  |  |
|  | *p* | .523 | **< .001** | .679 | — |  |  |  |  |  |  |
| PANAS-C negative affect | *rho* | -.020 | -.397 | -.154 | .500 | — |  |  |  |  |  |
|  | BF_10_ | 0.15 | > 100 | 0.49 | > 1000 | — |  |  |  |  |  |
|  | *p* | .860 | **< .001** | .183 | **< .001** | — |  |  |  |  |  |
| PANAS-C positive affect | *rho* | -.047 | .605 | .273 | -.381 | -.331 | — |  |  |  |  |
|  | BF_10_ | 0.16 | > 1000 | 4.81 | > 100 | 13.47 | — |  |  |  |  |
|  | *p* | .686 | **< .001** | **.016** | **< .001** | **.003** | — |  |  |  |  |
| PSWQ-C | *rho* | .207 | -.184 | -.039 | .439 | .300 | -.179 | — |  |  |  |
|  | BF_10_ | 1.06 | 0.63 | 0.16 | > 1000 | 9.55 | 0.59 | — |  |  |  |
|  | *p* | .071 | .108 | .737 | **< .001** | **.008** | .118 | — |  |  |  |
| PANAS-C-P negative affect (shortened) | *rho* | .102 | -.234 | -.022 | .191 | .165 | -.095 | .233 | — |  |  |
|  | BF_10_ | 0.24 | 1.22 | 0.16 | 0.69 | 0.49 | 0.21 | 1.06 | — |  |  |
|  | *p* | .405 | .053 | .860 | .116 | .176 | .435 | .054 | — |  |  |
| PANAS-C-P positive affect (shortened) | *rho* | -.216 | .321 | .232 | -.202 | -.177 | .136 | -.016 | -.132 | — |  |
|  | BF_10_ | 0.80 | 8.15 | 1.35 | 0.64 | 0.54 | 0.34 | 0.16 | 0.29 | — |  |
|  | *p* | .074 | **.007** | .055 | .096 | .146 | .264 | .897 | .281 | — |  |
| PSMFQ | *rho* | .011 | -.114 | .105 | .062 | .059 | < .001 | .194 | .573 | -.269 | — |
|  | BF_10_ | 0.16 | 0.29 | 0.24 | 0.18 | 0.18 | 0.16 | 0.59 | > 1000 | 2.53 | — |
|  | *p* | .926 | .353 | .389 | .612 | .629 | .999 | .110 | **< .001** | **.025** | — |

*Note.* Significant correlations highlighted in bold. *rho* = Spearman’s *rho*; BF_10_ = Bayes Factor in favour of the alternative hypothesis; SCWBS = Stirling Children’s Wellbeing Scale; CSMFQ = Child Shortened Mood and Feelings Questionnaire; PANAS-C = Positive and Negative Affect Schedule for Children; PSWQ-C = Penn State Worry Questionnaire for Children; PANAS-C-P = Positive and Negative Affect Schedule for Children – Parent’s version; PSMFQ = Parent Shortened Mood and Feelings Questionnaire.

# Correlations Between Carer-Reported Mood and Mind Wandering

**Table S3**

*Zero-Order Correlations for Carer-Reported Measures of Mood and Affect and Mind Wandering*

|  |  | Age | PANAS-C-P negative affect | PANAS-C-P positive affect | PSMFQ |
| --- | --- | --- | --- | --- | --- |
| Total mind wandering | *rho* | .083 | .129 | -.061 | .318 |
|  | BF_10_ | 0.18 | 0.20 | 0.17 | 2.41 |
|  | *p* | .476 | .290 | .617 | **.008** |
| Future mind wandering | *rho* | .107 | .250 | -.060 | .218 |
|  | BF_10_ | 0.36 | 2.68 | 0.20 | 0.51 |
|  | *p* | .355 | **.039** | .622 | .072 |
| Present mind wandering | *rho* | .002 | -.030 | .058 | .147 |
|  | BF_10_ | 0.14 | 0.18 | 0.17 | 1.64 |
|  | *p* | .989 | .809 | .636 | .229 |
| Past mind wandering | *rho* | .007 | -.045 | -.057 | .100 |
|  | BF_10_ | 0.14 | 0.16 | 0.16 | 0.16 |
|  | *p* | .951 | .716 | .646 | .418 |

*Note.* Significant correlations highlighted in bold. *rho* = Spearman’s *rho*; BF_10_ = Bayes Factor in favour of the alternative hypothesis; SCWBS = Stirling Children’s Wellbeing Scale; CSMFQ = Child Shortened Mood and Feelings Questionnaire; PANAS-C = Positive and Negative Affect Schedule for Children; PSWQ-C = Penn State Worry Questionnaire for Children; PANAS-C-P = Positive and Negative Affect Schedule for Children – Parent’s version; PSMFQ = Parent Shortened Mood and Feelings Questionnaire.

# Correlations Between Self-Reported Measures of Mood

**Table S4**

*Zero-Order Correlation Matrix for Age and Self-Reported Measures of Mood*

|  |  | 1 | 2 | 3 | 4 | 5 | 6 | 7 | 8 |
| --- | --- | --- | --- | --- | --- | --- | --- | --- | --- |
| 1. Age | *rho* | — |  |  |  |  |  |  |  |
|  | BF_10_ | — |  |  |  |  |  |  |  |
|  | *p* | — |  |  |  |  |  |  |  |
| 1. SCWBS | *rho* | -.125 | — |  |  |  |  |  |  |
|  | BF_10_ | 0.28 | — |  |  |  |  |  |  |
|  | *p* | .280 | — |  |  |  |  |  |  |
| 1. SCWBS (social desirability score) | *rho* | -.114 | .389 | — |  |  |  |  |  |
|  | BF_10_ | 0.22 | > 1000 | — |  |  |  |  |  |
|  | *p* | .324 | **< .001** | — |  |  |  |  |  |
| 1. CSMFQ | *rho* | .074 | -.445 | -.048 | — |  |  |  |  |
|  | BF_10_ | 0.17 | > 1000 | 0.24 | — |  |  |  |  |
|  | *p* | .523 | **< .001** | .679 | — |  |  |  |  |
| 1. PANAS-C negative affect | *rho* | -.020 | -.397 | -.154 | .500 | — |  |  |  |
|  | BF_10_ | 0.14 | 81.37 | 0.91 | 688.27 | — |  |  |  |
|  | *p* | .860 | **< .001** | .183 | **< .001** | — |  |  |  |
| 1. PANAS-C positive affect | *rho* | -.047 | .605 | .273 | -.381 | -.331 | — |  |  |
|  | BF_10_ | 0.14 | > 1000 | 14.70 | 38.22 | 5.08 | — |  |  |
|  | *p* | .686 | **< .001** | **.016** | **< .001** | **.003** | — |  |  |
| 1. PSWQ-C | *rho* | .207 | -.184 | -.039 | .439 | .300 | -.179 | — |  |
|  | BF_10_ | 0.38 | 0.69 | 0.21 | 154.17 | 9.30 | 0.42 | — |  |
|  | *p* | .071 | .073 | .737 | **< .001** | **.008** | .118 | — |  |
| 1. Negative mood composite | *rho* | .111 | -.820 | -.292 | .757 | .692 | -.731 | .350 | — |
|  | BF_10_ | 0.23 | > 1000 | 49.79 | > 1000 | > 1000 | > 1000 | 28.96 | — |
|  | *p* | .336 | **< .001** | **< .010** | **< .001** | **< .001** | **< .001** | **.002** | — |

*Note.* Significant correlations highlighted in bold. BF_10_ = Bayes Factor in favour of the alternative hypothesis; *rho* = Spearman’s *rho*; SCWBS= Stirling Children’s Wellbeing Scale; CSMFQ = Child Shortened Mood and Feelings Questionnaire; PANAS-C = Positive and Negative Affect Schedule for Children; PSWQ-C = Penn State Worry
